# Allelic variants of *OsHKT1;1* underlie the divergence between *indica* and *japonica* subspecies of rice (*Oryza sativa*) for root sodium content

**DOI:** 10.1371/journal.pgen.1006823

**Published:** 2017-06-05

**Authors:** Malachy T. Campbell, Nonoy Bandillo, Fouad Razzaq A. Al Shiblawi, Sandeep Sharma, Kan Liu, Qian Du, Aaron J. Schmitz, Chi Zhang, Anne-Aliénor Véry, Aaron J. Lorenz, Harkamal Walia

**Affiliations:** 1 Department of Agronomy and Horticulture, University of Nebraska-Lincoln, Lincoln, Nebraska, United States of America; 2 Laboratoire de Biochimie et Physiologie Moléculaire des Plantes, Unité Mixte de Recherche Centre National de la Recherche Scientifique (5004)/Institut National de la Recherche Agronomique (388)/SupAgro/Université Montpellier, Montpellier, France; 3 School of Biological Sciences, University of Nebraska-Lincoln, Lincoln, Nebraska, United States of America; University of California, San Diego, UNITED STATES

## Abstract

Salinity is a major factor limiting crop productivity. Rice (*Oryza sativa*), a staple crop for the majority of the world, is highly sensitive to salinity stress. To discover novel sources of genetic variation for salt tolerance-related traits in rice, we screened 390 diverse accessions under 14 days of moderate (9 dS·m^-1^) salinity. In this study, shoot growth responses to moderate levels of salinity were independent of tissue Na^+^ content. A significant difference in root Na^+^ content was observed between the major subpopulations of rice, with *indica* accessions displaying higher root Na^+^ and *japonica* accessions exhibiting lower root Na^+^ content. The genetic basis of the observed variation in phenotypes was elucidated through genome-wide association (GWA). The strongest associations were identified for root Na^+^:K^+^ ratio and root Na^+^ content in a region spanning ~575 Kb on chromosome 4, named *Root Na*^*+*^ *Content 4* (*RNC4*). Two Na^+^ transporters, *HKT1;1* and *HKT1;4* were identified as candidates for *RNC4*. Reduced expression of both *HKT1;1* and *HKT1;4* through RNA interference indicated that *HKT1;1* regulates shoot and root Na^+^ content, and is likely the causal gene underlying *RNC4*. Three non-synonymous mutations within HKT1;1 were present at higher frequency in the *indica* subpopulation. When expressed in *Xenopus* oocytes the *indica-*predominant isoform exhibited higher inward (negative) currents and a less negative voltage threshold of inward rectifying current activation compared to the *japonica*-predominant isoform. The introduction of a 4.5kb fragment containing the HKT1;1 promoter and CDS from an *indica* variety into a *japonica* background, resulted in a phenotype similar to the *indica* subpopulation, with higher root Na^+^ and Na^+^:K^+^. This study provides evidence that *HKT1;1* regulates root Na^+^ content, and underlies the divergence in root Na^+^ content between the two major subspecies in rice.

## Author summary

Despite intensive research, few genes have been identified that underlie natural variation for salinity responses in rice. In this study, we used a rice diversity panel for genome wide association mapping to identify *HKT1;1* as a factor regulating Na^+^ distribution. Within the rice diversity panel we observed higher Na^+^ levels in root tissue in the *indica* subpopulation compared to *japonica* accessions. Three non-synonymous variants were identified within HKT1;1 that were associated with altered Na^+^ accumulation in root tissue, and displayed contrasting frequencies between *indica* and *japonica* subspecies. The introduction of HKT1;1 from an *indica* accession that contained the three non-synonymous variants into a *japonica* background resulted in a phenotype similar to that exhibited by the *indica* subpopulation. This work suggests that these allelic variants are likely responsible for the higher root Na^+^ observed in *indica* accessions. This study has identified a genetic resource for modifying Na^+^ content rice, and provides evidence that *HKT1;1* underlies the divergence between *indica* and *japonica* subspecies in root Na^+^ content.

## Introduction

Salinity is a widespread limitation for agricultural productivity, especially for irrigated agriculture and coastal lowlands prone to seawater ingress [1,2]. By definition, salinity occurs when there is a high concentration of soluble salts in soil [3]. More than 800 million hectares worldwide is affected by salt, which accounts for 6% of the total land area [3]. Besides natural causes such as rising sea levels during the dry and wet cropping seasons, the poor quality of irrigation water and improper drainage, also collectively increases soluble salt concentration in the root zone [2,4].

Rice (*Oryza sativa* L.) is one of the most important crop species and is a staple food for more than half of the world’s population. Salinity is a major impediment to increasing production in many rice growing regions, including temperate and tropical environments, around the world [5,6]. Rice is the most salt-sensitive species among major cereal crops [3]. The susceptibility of rice to salinity stress varies with growth stages [7,8]. Rice is less sensitive to saline conditions at germination, active tillering and maturity stage [7,9,10]. Vegetative growth during the early seedling stage is highly sensitive to saline conditions, and often translates to reduced stand density in salt-affected fields [11,12]. Some rice varieties are most sensitive to salt stress during early tillering and panicle initiation stages of growth [8]. This developmentally-dependent salt-sensitivity, in context of yield reduction, was associated with a significant decrease in tiller number per plant, spikelet number per panicle, fertility, panicle length and primary branches per panicle [7,8,13,14].

Despite the overall high salt-sensitivity of rice, several studies have demonstrated that considerable natural variation for salinity tolerance exists in rice germplasm [15,16]. Traditional landraces or cultivars such as ‘Pokkali’, ‘Nona Bokra’, ‘Cheriviruppu’ and ‘SR26B’ have originated or have been selected in coastal regions and are more tolerant to saline conditions [5,12,17,18]. Quantitative trait loci (QTL) underlying salinity tolerance have undergone intensive investigations [16,18–24]. Although many QTL have been identified across the rice genome, the most well-characterized QTL is *Saltol/SKC1*, which harbors *HKT1;5*, on the short arm of chromosome 1 [18,20–22]. The *SKC1* gene (*HKT1;5*) was subsequently cloned from a salt-tolerant *indica* landrace, ‘Nona Bokra’, and encodes a Na^+^ transporter that regulates shoot Na^+^:K^+^ homeostasis during salt stress [25].

Salinity tolerance is a complex polygenic trait, and several physiological mechanisms, including tissue tolerance, sodium exclusion, osmotic stress tolerance, and tissue-specific sodium sequestration can be utilized for improving salinity tolerance [3]. While many QTL have been reported for salinity tolerance in rice, few studies have identified the causal genes and confirmed the importance of these resources for improving salinity tolerance. Hence, the genetic resources (QTL and genes) available to rice breeders for improving salt tolerance are limited. Identification of loci that regulate salt accumulation and/or distribution will enable the introgression of favorable genic combinations and greatly accelerate the development of robust salt-tolerant rice varieties.

Genetic variation within the rice germplasm collection can be utilized to identify important loci controlling variation for salinity tolerance through genome-wide association (GWA) analysis, which provides greater mapping resolution and evaluates greater allelic diversity compared to linkage mapping strategies [16,24,26,27]. In this study, we used GWA to investigate the genetic architecture of salinity tolerance using the Rice Diversity Panel 1 (RDP1) [28–30]. RDP1 is comprised of 421 accessions collected from 85 countries and was developed to identify alleles associated with morphological, physiological and agronomic traits [28–30]. RDP1 captures much of the diversity in the rice germplasm collection worldwide [28–30]. We used several quantitative measures to characterize the rice diversity panel for physiological and morphological responses to salinity stress. Here, we show that allelic variants of a sodium transporter (*HKT1;1*) underlie natural variation for root Na^+^ content in rice. Using a multifaceted approach, we demonstrate that variants within HKT1;1 alter Na^+^ transport and can explain the basis of divergence in root Na^+^ content between the *indica* and *japonica* subspecies of cultivated rice.

## Results

To assess the degree of natural variation for salinity tolerance associated traits in rice, a 9 dS·m^-1^ (~90 mM NaCl) salt stress was imposed gradually over a period of four days (in four increments of 20–30 mM) to two-week old rice seedlings. A rice diversity panel, consisting of 390 rice accessions (383 from RDP1 and seven check varieties), was scored for ten phenotypic traits at the end of a two-week 9 dS·m^-1^ stress period (the plants were 28 days old). The ten traits recorded were root biomass (control and salt conditions), shoot biomass (control and salt conditions), root and shoot Na^+^ content, root and shoot K^+^ content, and root and shoot Na^+^:K^+^ (S1 File). To control for inherent differences in growth rate between lines, we expressed the saline-induced growth response for each accession as the ratio of biomass in saline conditions over biomass in control conditions. Broad sense heritability for the eight phenotypic traits ranged from 0.32 for root K^+^ content and 0.83 for shoot biomass in control conditions (S1 Table).

### Rice subpopulations exhibit inherent differences in ion homeostasis

To examine the relationships between each of the eight traits, Pearson correlation analysis was performed across all accessions. No significant relationship was observed between shoot biomass and ion traits (S3 Table). Moreover, root and shoot ion content showed no significant relationship when the analysis was performed with all accessions (S3 Table). Due to the deep population structure in rice, correlation analysis was also performed for each of the five major subpopulations in RDP1 (here, admixed accessions were considered a separate subpopulation; S4–S8 Tables) [29,31]. Root growth response (the ratio of root biomass in salt to control) showed a weak negative correlation with shoot Na^+^:K^+^ in *admix* and *tropical japonica* (*trj*) accessions (S4 and S5 Tables, respectively). In *trj*, *aus*, and *tej* subpopulations significant, albeit weak, positive correlations were observed between shoot Na^+^ and root Na^+^:K^+^ (S5–S7 Tables).

**Fig 1:**
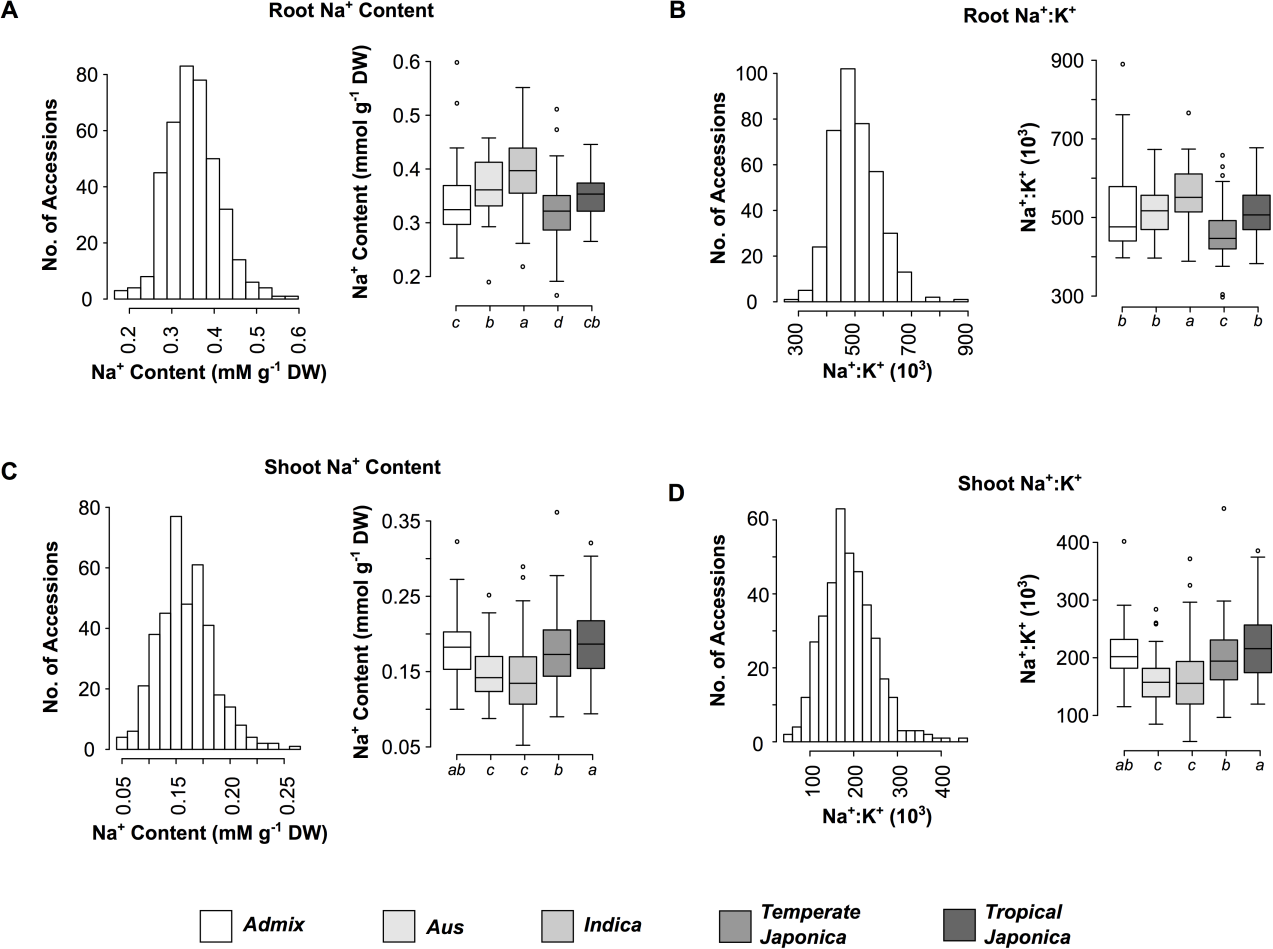
Phenotypic variation and distribution of root and shoot Na^+^ content and Na^+^:K^+^ among the five subpopulations of RDP1. (A) Root Na^+^ content, (B) root Na^+^:K^+^, (C) shoot Na^+^ content, and (D) shoot Na^+^:K^+^. Accessions were assigned to each subpopulation according to Famoso *et al* [29]. Boxplots with the same letter indicate no significant difference as determined using ANOVA (*p* < 0.05). *Aromatic* accessions were excluded from the analysis due to low *n*.

Comparisons between each of the subpopulations showed significant differences for shoot and root Na^+^, K^+^ and Na^+^:K^+^ (Fig 1). *Indica* accessions exhibited significantly higher root Na^+^ content and Na^+^:K^+^ compared to the other four subpopulations (Fig 1A and 1B). Significantly lower shoot Na^+^ and Na^+^:K^+^ were observed in *indica* and *aus* subpopulations compared to *temperate japonica* (*tej*), *tropical japonica* (*trj*) and *admix* accessions (Fig 1C and 1D). These results suggest that there are inherent differences in root and shoot ion homeostasis between subpopulations, with *indica* accessions generally displaying higher root Na^+^ and Na^+^:K^+^, and *indica* and *aus* accessions exhibiting lower shoot Na^+^ content and Na^+^:K^+^.

### Genome-wide association mapping identifies a major QTL on chromosome 4 for root Na^+^ and Na^+^:K^+^

**Fig 2:**
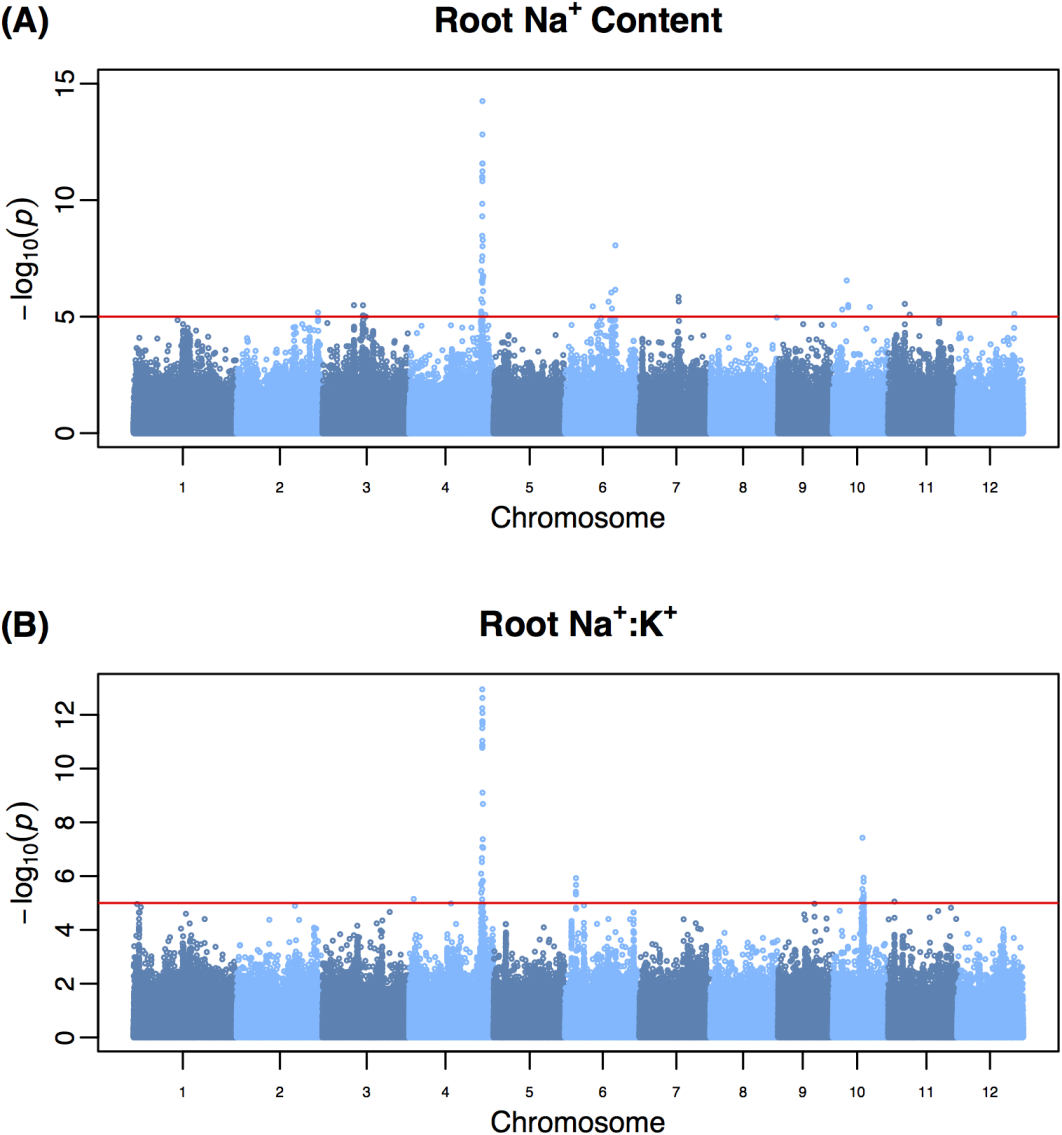
Genome-wide association analysis of Na^+^ content and Na^+^:K^+^ in root tissue. (A) Root Na^+^ content, (B) root Na^+^:K^+^. Genome-wide association (GWA) was performed using a mixed model that accounted for population structure and relatedness between accessions of RDP1 using 365 accessions of RDP1 and 397,812 SNPs. For each trait the least squares mean was used as the dependent variable The red horizontal line indicates a statistical significance threshold of *p* < 10^−5^, and was determined using the M_eff_ method with an experiment-wise error rate of 0.05 [63].

To identify loci associated with salt tolerance-related phenotypes, GWA mapping was conducted using 397,812 SNPs and eight salinity-related phenotypes collected on 365 rice accessions (Fig 2; S1–S4 Figs) [32]. A linear mixed model implemented in EMMA was used for the association analysis [33]. A total of 90 highly significant QTL (245 SNPs; *p* < 10^−5^) were identified for salinity-related traits with the strongest associations detected for root Na^+^ content followed by root Na^+^:K^+^ (Fig 2A and 2B, respectively). A region located at ~30.6 Mb on chromosome 4 was found to have the largest effect and explained 15% of the phenotypic variation beyond that explained by population structure for root Na^+^:K^+^ (S2 File). An additional 25% of the phenotypic variance for root Na^+^:K^+^ was explained by population structure suggesting that this trait may be heavily influenced by the differences between the major subpopulations in rice.

For each trait, the number of significant QTL ranged from 3–24, with the highest number of QTL identified for root biomass ratio (24 QTL). Many of these QTL had small effects, explaining ~4.7–7.5% of phenotypic variation for root growth response. These results indicate a polygenetic architecture for root growth responses to salinity. A large number of QTL with minor effects (explaining < 7% phenotypic variation) were identified for shoot Na^+^ content and Na^+^:K^+^, suggesting a polygenic architecture for these traits in rice. This trend was observed for all traits, with the exception of root Na^+^ and Na^+^:K^+^, suggesting that salinity tolerance in terms of growth and shoot ion homeostasis in rice is regulated by many loci with small effects. Twenty QTL were commonly detected for two or more traits. Shoot Na^+^ and Na^+^:K^+^ showed the largest number of shared QTL (12 QTL), however much of this similarity is likely driven by the strong phenotypic and genetic correlation observed between these traits within tissues (Table 1; S2 Table).

**Table 1:** Phenotypic and genetic correlation of root and shoot ion traits in RDP1.

Trait	Root Na^+^	Root K^+^	Root Na^+^:K^+^	Shoot Na^+^	Shoot K^+^	Shoot Na^+^:K^+^
**Root Na**^**+**^	1.00	0.25	0.81	0.06	0.11	-0.07
**Root K**^**+**^	0.47	1.00	-0.36	-0.21	0.09	-0.20
**Root Na**^**+**^**:K**^**+**^	0.66	-0.32	1.00	0.12	0.00	0.11
**Shoot Na**^**+**^	0.00	-0.09	0.06	1.00	-0.17	0.96
**Shoot K**^**+**^	0.14	0.14	0.04	-0.13	1.00	-0.41
**Shoot Na**^**+**^**:K**^**+**^	-0.02	-0.12	0.06	0.96	-0.35	1.00

The lower diagonal represents the phenotypic Pearson correlation coefficients, while the upper diagonal represents the genetic correlation coefficients (n = 383). Here, the genetic correlation between traits accounts for population structure and relatedness between accessions of RDP1.

### *HKT1;1* regulates Na^+^ homeostasis during the early tillering stage

**Fig 3:**
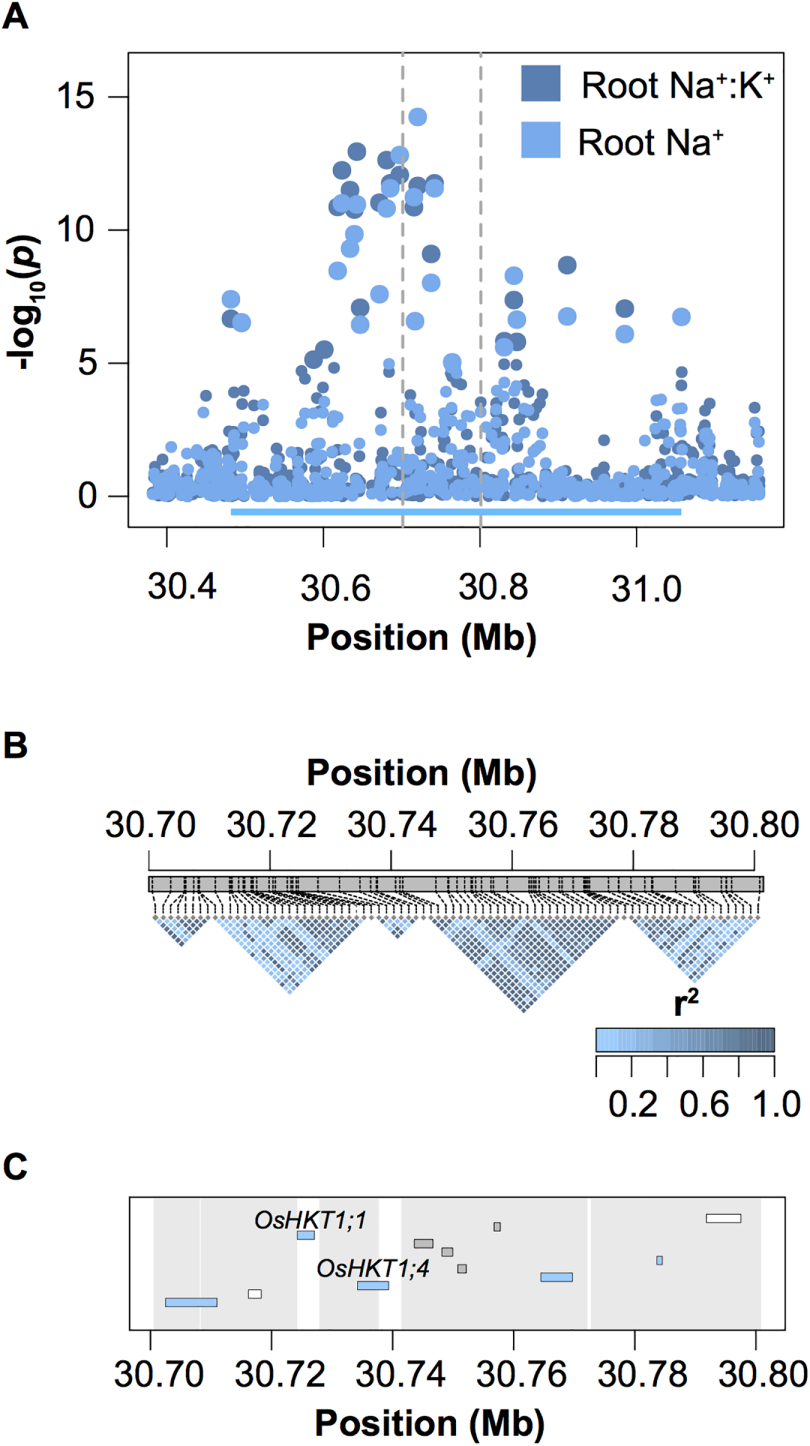
Genetic characterization of *RNC4*. (A) Regional manhattan plot summarizing GWA analysis of root Na^+^ and root Na^+:^K^+^. The region defining *RNC4* is indicated with the cyan bar. (B) LD plots for a subset of five haplotype blocks within *RNC4*. The vertical broken gray lines in A indicate the region characterized by haplotype blocks. The genes present in this region are illustrated in C. Genes encoding transposable elements are highlighted in the gene track in gray, while those encoding expressed proteins are highlighted in white. The regions defined by each block are indicated in gray.

The most significant QTL for root Na^+^ content and Na^+^:K^+^, named *Root Na*^*+*^ *Content 4* (*RNC4*), spans a region of ~575 Kb (30,481,871–31,057,205) on chromosome 4 (Fig 3A). To characterize this region further and identify candidate genes that may be underlying natural variation for this trait, this region was segmented into haplotype blocks and the contributions of each block to root Na^+^ content and Na^+^:K^+^ were determined using ANOVA. A total of 36 blocks were identified in this 575 Kb region (S5–S8 Figs). A single 9.7 Kb block from 30,727,920–30,737,580 bp was found to have the largest contribution to root Na^+^ and Na^+^:K^+^, with approximately 16% of phenotypic variation explained for root Na^+^ and 17.5% explained for root Na^+^:K^+^ (Fig 3B; Table 2). The region spanning from the 5’ boundary of block 2 to the 5’ boundary of block 3 harbored only two genes, both of which were annotated as sodium transporters, *HKT1;1* and *HKT1;4* (LOC_Os04g51820 and LOC_Os04g51830 respectively; Fig 3C).

**Table 2:** Proportion of variation explained by a subset of the haplotype blocks within *RNC4*.

		Root Ion			Shoot Ion		
Block	Position (bp)	Na^+^	K^+^	Na^+^:K^+^	Na^+^	K^+^	Na^+^:K^+^
1	30,700,577–30,708,292	0.013	0.049	0.027	0.013	0.058	0.012
2	30,708,321–30,724,332	0.045	0.036	0.054	0.035	0.066	0.040
3	30,727,920–30,737,580	0.160	0.024	0.175	0.037	0.029	0.032
4	30,740,743–30,771,971	0.012	0.123	0.069	0.145	0.094	0.125
5	30,772,483–30,795,385	0.060	0.033	0.079	0.037	0.050	0.036

Haplotype blocks were determined using the 4Gamete rule in Haploview with a recombination threshold of > 2%. Analysis of variance (ANOVA) was used to estimate proportion of phenotypic variance accounted for by each block after adjusting for population structure effects.

**Fig 4:**
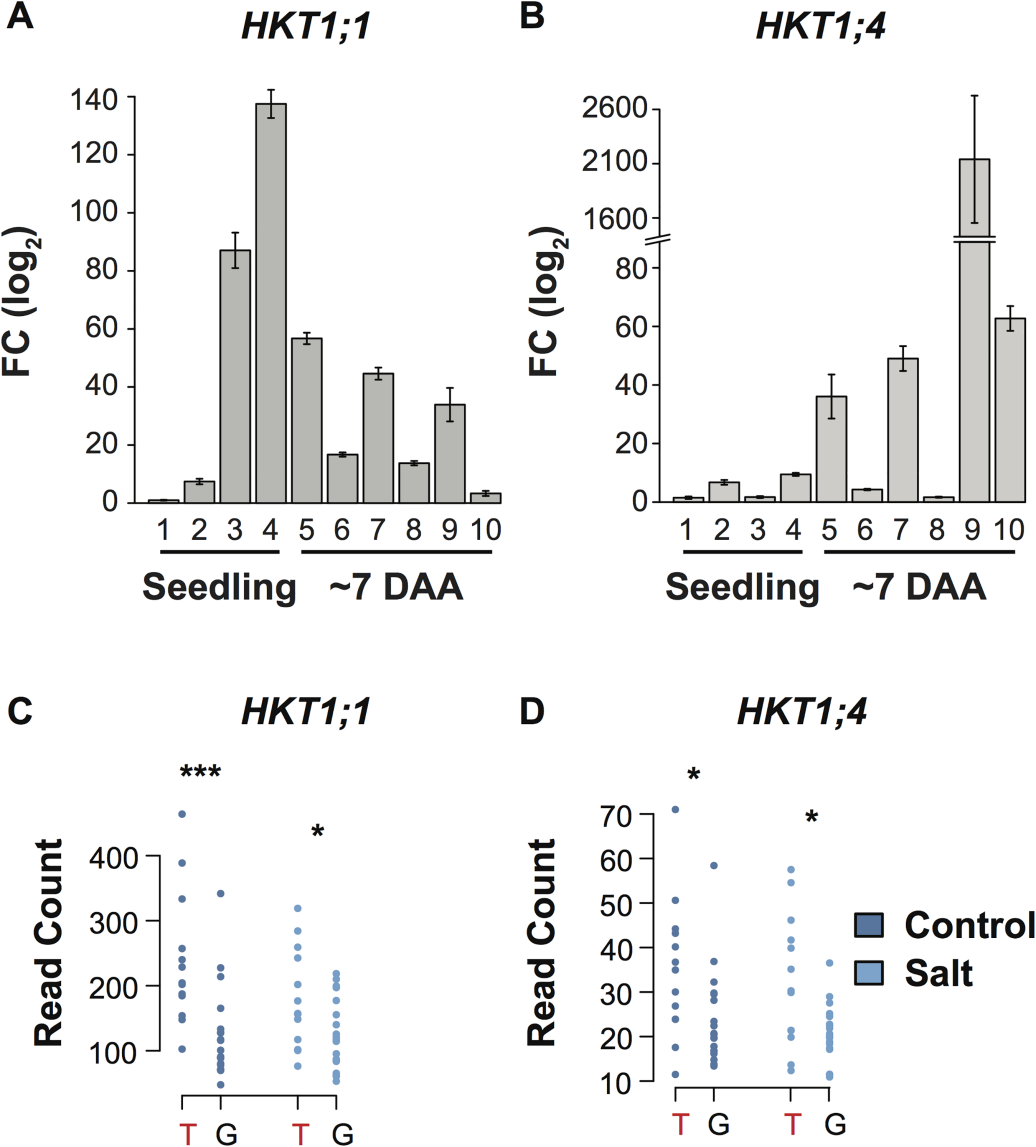
Characterization of HKT1;1 and HKT1;4 expression. Expression profiles of *HKT1;1* (A) and *HKT1;4* (B) in seeding and mature plants. Numbers below each of the bars indicate different tissues as follows: 1: seedling root, 2: seedling shoot, 3: blade of newest fully expanded leaf in seedling (leaf 3), 4: sheath of newest fully expanded leaf in seedling (leaf 3), 5: penultimate leaf sheath in mature plant, 6: penultimate leaf blade in mature plant, 7: flag leaf sheath in mature plant, 8: flag leaf blade in mature plant, 9: culm of mature plant and 10: panicle of mature plant. DAA: days after anthesis. (C, D) Dot plots comparing the expression of *HKT1;1* (C) and *HKT1;4* (D) between allelic groups at SNP-4-30535352 in control and saline conditions. The minor allele genotype, which displays higher root Na^+^ content, is indicated by red text. A Mann-Whitney test was performed within each treatment to determine differences between the two groups with asterisks indicating significance as determined using a one-way ANOVA: ***: *p* < 0.001; *: *p* < 0.05.

To further characterize *HKT1;1* and *HKT1;4*, the expression patterns of both genes were examined in twelve tissues at three developmental time points (early seedling, early tillering and anthesis). The expression of both *HKT1;1* and *HKT1;4* were higher in leaf tissue compared to root tissue during the seedling stage (Fig 4, S9 Fig). However, the expression of *HKT1;1* and *HKT1;4* within aerial tissues differed across developmental stages. *HKT1;1* was highly expressed in the leaf blade and leaf sheath during the early seedling stage (Fig 4A). *HKT1;4*, on the other hand displayed the highest expression during reproductive stage, specifically in culm tissue at ~7 days after anthesis (Fig 4B).

To examine whether transcript abundance may be a component of the phenotypic differences observed between allelic groups at *RNC4*, RNA sequencing was performed on shoot tissue of 32 accessions in control and saline conditions, and the expression of both genes was compared between allelic groups at *RNC4* (Fig 4C and 4D; S3 File). For both genes, accessions that showed higher root Na^+^ content (T allele at SNP-4-30535352), also showed higher expression in both control and saline conditions compared to accessions with low root Na^+^ content (G allele at SNP-4-30535352). The expression of *HKT1;1* was approximately 92% higher in high root Na^+^ lines in control conditions compared to low root Na^+^ lines, while a 44% higher expression was observed in saline conditions (Fig 4C). While the overall expression level was much lower for *HKT1;4* compared to *HKT1;1*, a similar trend in gene expression was also observed between the two allelic groups of *HKT1;4* (Fig 4D). A 46% and 57% higher expression was observed in lines with high root Na^+^ content compared to lines with low root Na^+^ content in control and saline conditions, respectively (Fig 4D). These results suggest that differences in expression of *HKT1;1* and/or *HKT1;4* may be a component underlying variation in root Na^+^ content at *RNC4*.

**Fig 5:**
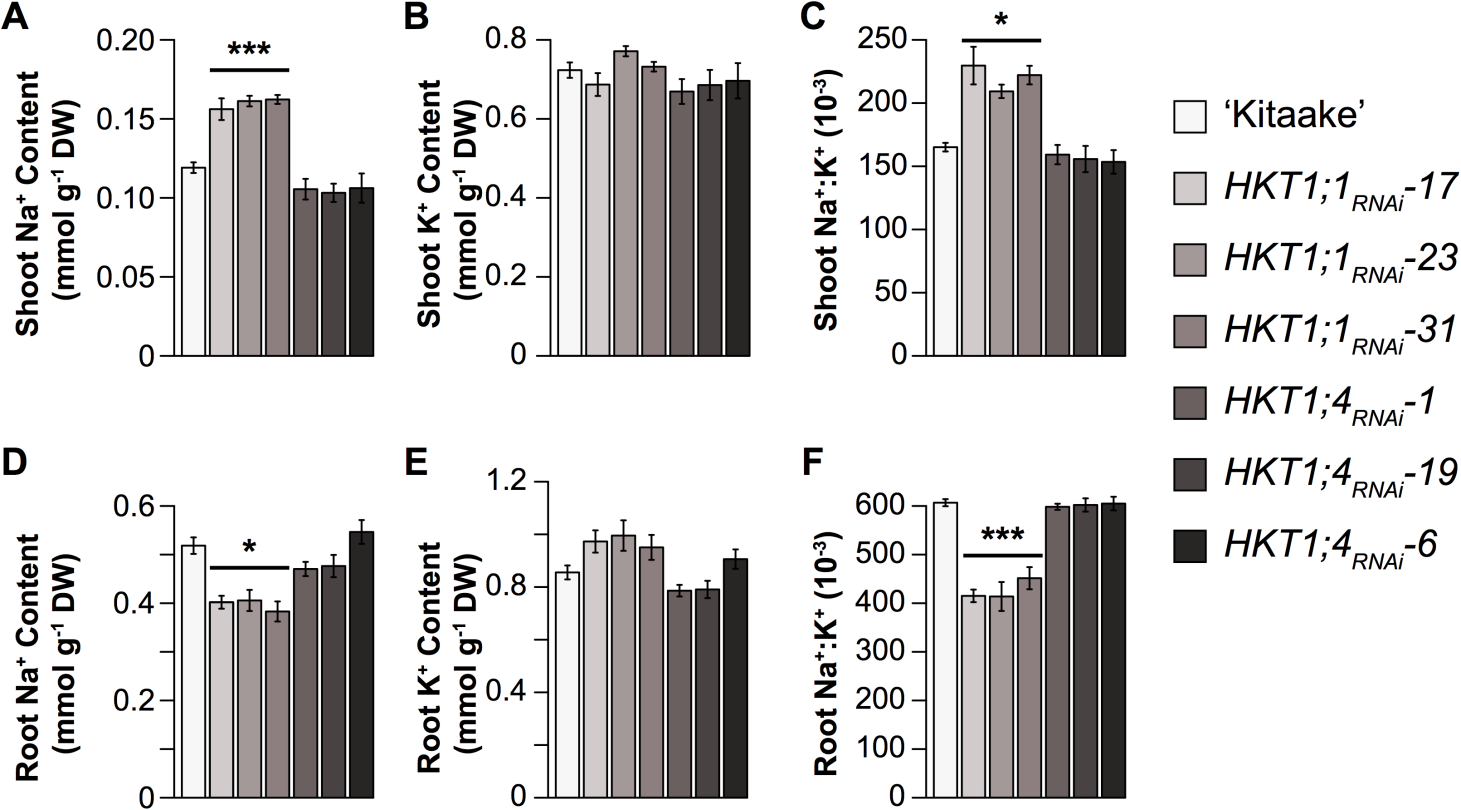
**Root (A-C) and shoot (D-F) ion content of *HKT1;1* and *HKT1;4* RNAi plants.** Asterisks indicate significance as determined using Tukey’s HSD: ***: *p* < 0.001; *: *p* < 0.05. Pairwise comparisons were made between lines within each treatment. Error bars represent standard error of the mean where n = 12–20 plants.

To determine if these two HKTs within *RNC4* regulate Na^+^ content during salinity stress at the early tillering stage, three independent RNA-interference (*RNAi*) lines were generated for both genes. Transcript levels in the leaf tissue was reduced by approximately 2.9–6.2 and 2–2.2 fold in *HKT1;4*_*RNAi*_ and *HKT1;1*_*RNAi*_ lines compared to wild-type (*WT*) ‘Kitaake’, respectively (S10 Fig). A 9 dS·m^-1^ (~90 mM NaCl) was gradually imposed at 10 DAT for 14 days to replicate the stress treatment for the large-scale screening. Reduced expression of *HKT1;1* had severe phenotypic effects on shoot and root ion homeostasis as well as shoot and root growth under salinity. Shoot Na^+^ and Na^+^:K^+^ were 31–41% and 27–41% higher, respectively, in *HKT1;1*_*RNAi*_ lines compared to *WT* (*p* < 0.0001, *p* < 0.05 respectively; Fig 5A–5C). A 21–27% reduction in root Na^+^ was observed in *HKT1;1*_*RNAi*_ and 31–33% lower root Na^+^:K^+^ was observed in *HKT1;1*_*RNAi*_ compared to *WT* (*p* < 0.05 and *p* < 0.0001, respectively; Fig 5D–5F). In *RNAi* plants, shoot and root growth was reduced by 44–55% and 78–72% respectively in salt treated plants relative to those in control conditions, while in *WT* a 26% and 45% reduction in shoot and root growth, respectively was observed in *WT* plants (S11 Fig). No differences were observed between *HKT1;4*_*RNAi*_ and *WT* plants (Fig 5, S11 Fig). These results suggest that *HKT1;1* may influence the shoot and root Na^+^ content during the early tillering stage, and is likely the causal gene underlying *RNC4*.

### Allelic variants of HKT1;1 alter Na^+^ transport

**Fig 6:**
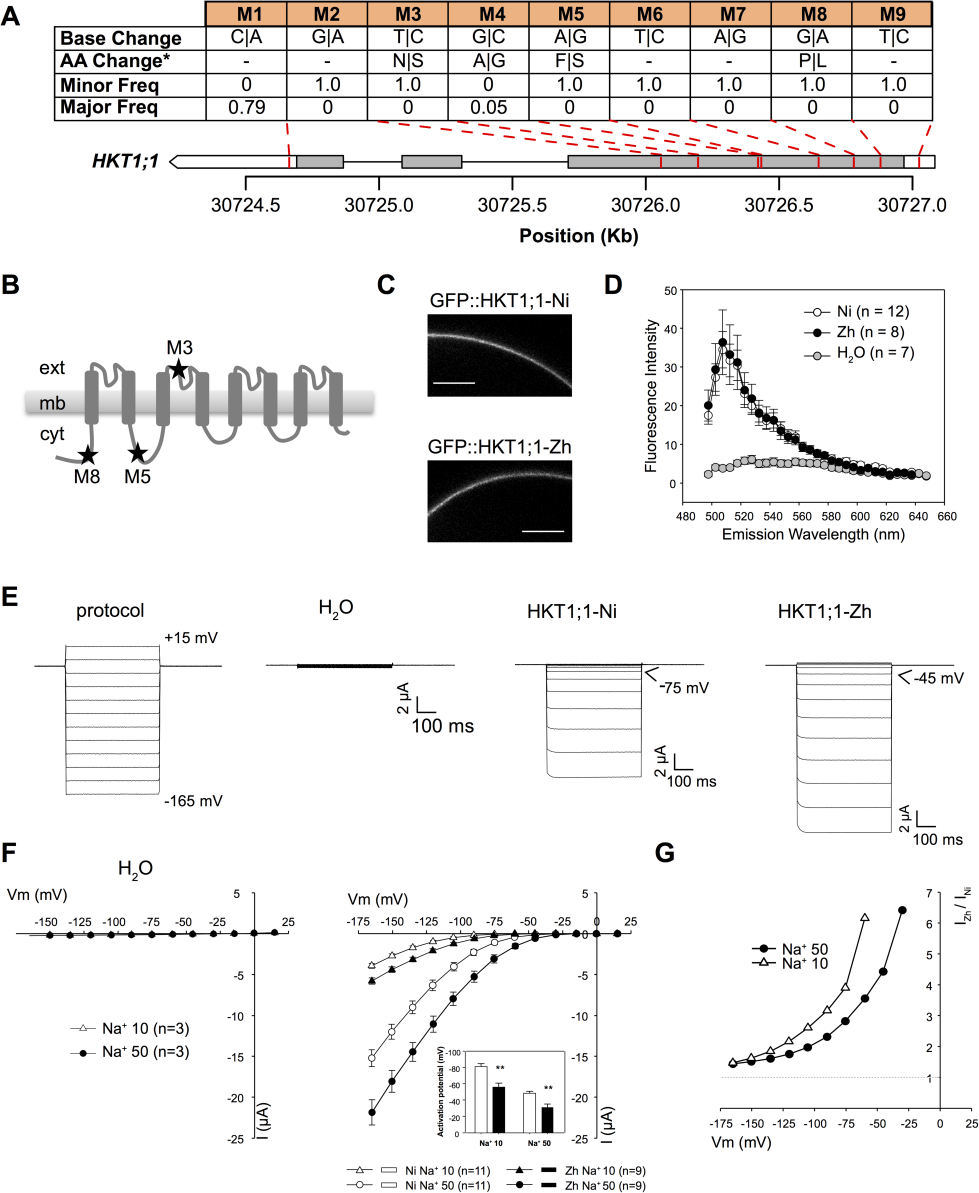
Characterization of high and low root Na^+^ isoforms of HKT1;1 in *Xenopus* oocytes. (A) Genetic variants within the ORF of *HKT1;1*. “Minor Freq” and “Major Freq” indicate the frequency of the alternate allele in the major (*n* = 19) and minor (*n* = 13) allelic groups at SNP-4-30535352. “AA change” indicates the resulting changes in protein sequence. Synonymous mutations are indicated by “-”. The grey bars represent exons while the white bars represent the 3’ and 5’ UTRs. (B) Secondary structure of OsHKT1;1 polypeptide showing the position of AA changes, as exemplified between ‘Nipponbare’ and ‘Zhenshan 2’ variants. (C, D) Comparison of OsHKT1;1-Ni and–Zh targeting to the oocyte membrane by confocal imaging of GFP-tagged transporters. (C) Representative images of oocytes expressing Ni (top) or Zh (bottom) transporters. Emitted fluorescence was collected between 505 and 510 nm. Scale bar: 100 μm. (D) Comparison of fluorescence intensity spectra at the membrane in water-injected oocytes (control) and in oocytes expressing either of the HKT1;1 variants. Data are means ± SE. (E) Voltage-clamp protocol and corresponding representative current traces recorded in control oocytes or oocytes expressing the HKT1;1 variants, in 50 mM Na-glutamate-containing bath solution. (F) Current-voltage (I-V) relationships in control oocytes (left) and in HKT1;1-Ni or -Zh-expressing oocytes (right), in either 10 or 50 mM Na-glutamate-containing bath solutions. Data are means ± SE. Insert: Activation potential of HKT1;1-Ni or -Zh currents. Asterisks indicate significant difference in activation potential as determined using Student’s t test: **: *p* < 0.005. (G) HKT1;1-Zh to HKT1;1-Ni mean current ratio at varying membrane potentials, determined from I-V data shown in (F). Shown data in (E to G) were obtained in a single oocyte batch and are representative of three experiments performed in different oocyte batches.

To determine whether there were sequence differences between allelic groups at *RNC4*, sequencing data was mined for variants in *HKT1;1* (S4 File). Nine variants were detected in the coding region of *HKT1;1* with four SNPs resulting in non-synonymous amino acid substitutions in *HKT1;1* (Fig 6; S12 Fig) [34]. Of the nine variants, only M4 displayed a significant deviation from the expected frequency in the minor allelic group, indicating that it is unlikely to be important for the high root Na^+^ phenotype exhibited by accessions in the minor allelic group (Pearson’s chi squared test, *p* < 1.26 x 10^−5^). The remaining three non-synonymous mutations (M3, M5 and M8) were detected in thirteen accessions all belonging to the minor allelic group, which is characterized by high root Na^+^ content, at the most significant SNP for root Na^+^ content (SNP-4-30535352). The higher frequency of these three non-synonymous mutations observed in minor allele accessions (T) suggests that allelic variation in *HKT1;1* could be a component in the genetic basis of the observed difference in root Na^+^ content between major and minor alleles. No sequence differences in *HKT1;4* were observed between allelic groups at *RNC4*.

To characterize the biophysical properties of the two major isoforms identified between allelic groups at *RNC4*, *HKT1;1* was isolated from two representative accessions, ‘Nipponbare’ and ‘Zhenshan 2’, which have the reference and the three non-synonymous mutations at the three locations (M3, M5 and M8), respectively (S12 Fig). At the transporter structure level, two non-synonymous SNPs (M8 and M5) lead to amino acid substitutions in cytosolic regions of HKT1;1: proline to leucine within the N-terminal cytosolic region, phenylalanine to serine in the cytosolic loop between the first and second transmembrane segment-pore region-transmembrane segment (MPM) domains (Fig 6A and 6B; S12 Fig). The third non-synonymous SNP results in an asparagine to serine substitution in the external part of the pore-forming region of the second MPM (Fig 6A and 6B; S12 Fig). Functional analysis was performed by voltage-clamp electrophysiology using *Xenopus* oocytes for the two variants of HKT1;1 (Fig 6). The amount of expressed transporters targeted to the oocyte membrane was similar for the two variants, as indicated by the mean GFP fluorescence intensity emitted by either of the tagged transporters at the membrane (Fig 6C and 6D). In agreement with previous reports, both isoforms of HKT1;1 displayed low affinity, high Na^+^ *versus* K^+^ selectivity, inward rectifying activity and no time-dependent kinetics (Fig 6E; S13 and S14 Figs) [34]. However, the two allelic variants displayed considerable differences in Na^+^ transport activity. The variant from the accessions with high root Na+, HKT1;1-Zh, exhibited higher inward (negative) currents compared to that from ‘Nipponbare’, HKT1;1-Ni (Fig 6E and 6F), essentially due to a less negative voltage threshold of inward rectifying current activation by 20–25 mV in all ionic conditions (Fig 6E and 6F, S13 and S14 Figs). This latter feature was especially expected to favor transport activity of HKT1;1-Zh compared to HKT1;1-Ni during salinity stress where the high concentration of Na^+^ in the apoplast results in a depolarization of the plasma membrane [35,36]. Thus, at a weak negative voltage, the current could be more than six-fold higher in HKT1;1-Zh, compared to HKT1;1-Ni (Fig 6G).

**Fig 7:**
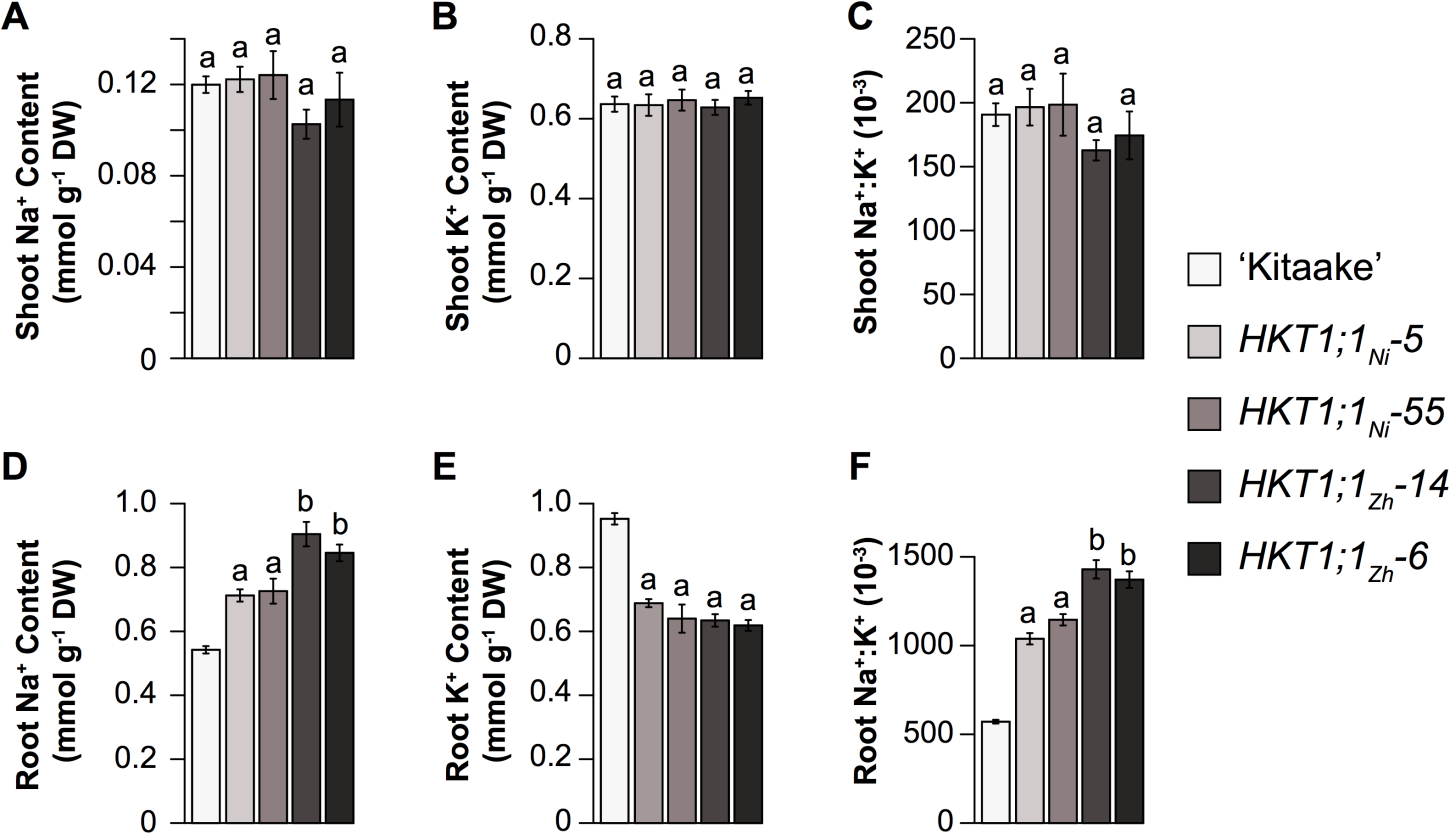
**Shoot (A-C) and root (D-F) ion content for *HKT1;1* native overexpression lines.** Statistical significance was determined using Tukey’s HSD test between each line within treatments. Bars with the same letters indicate no significant difference (*p* < 0.05). Error bars represent standard error of the mean where n = 12–18 plants.

To determine if these differences in transport activity have physiological effects *in vivo*, native overexpression lines were generated for each variant (*HKT1;1*_*Ni*_, *HKT1;1*_*Zh*_). A ~4.3 kb genomic region was isolated from ‘Nipponbare’ and ‘Zhenshan 2’, which included the entire CDS of *HKT1;1* and a 1.9 kb promoter, and was expressed in ‘Kitaake’. The endogenous *HKT1;1* in ‘Kitaake’, at the protein level, is identical to HKT1;1-Ni, and thus lacks the three non-synonymous variants. Two independent transformants for each variant (*HKT1;1*_*Ni*_, *HKT1;1*_*Zh*_), each containing only a single copy of the transgene, were evaluated under a 9 dS·m^-1^ salt stress for a period of two-weeks. The expression of *HKT1;1*_*Zh*_ resulted in an increase in root Na^+^ and Na^+^:K^+^ compared to *HKT1;1*_*Ni*_, while no differences were observed between variants for root K^+^ (Fig 7). A considerable increase in both root Na^+^ and Na^+^:K^+^, as well as a reduction in root K^+^ was observed in both native overexpression lines (*HKT1;1*_*Ni*_ and *HKT1;1*_*Zh*_) compared to ‘Kitaake’, which is opposite to the root phenotype observed in the *HKT1;1*_*RNAi*_ lines (Fig 7). However, expression under the native promoter had no effects on shoot Na^+^ or Na^+^:K^+^ (Fig 7). Together, these results provide further evidence that *HKT1;1* is responsible for the higher root Na^+^ phenotype, and that the difference in Na^+^ content between the allelic groups at *RNC4* is likely due to functional differences in Na^+^ transport by *HKT1;1* alleles, with the three non-synonymous SNPs in *HKT1;1-Zh* resulting in higher Na^+^ transport activity.

### Origins of HKT1;1 variants in cultivated rice

**Fig 8:**
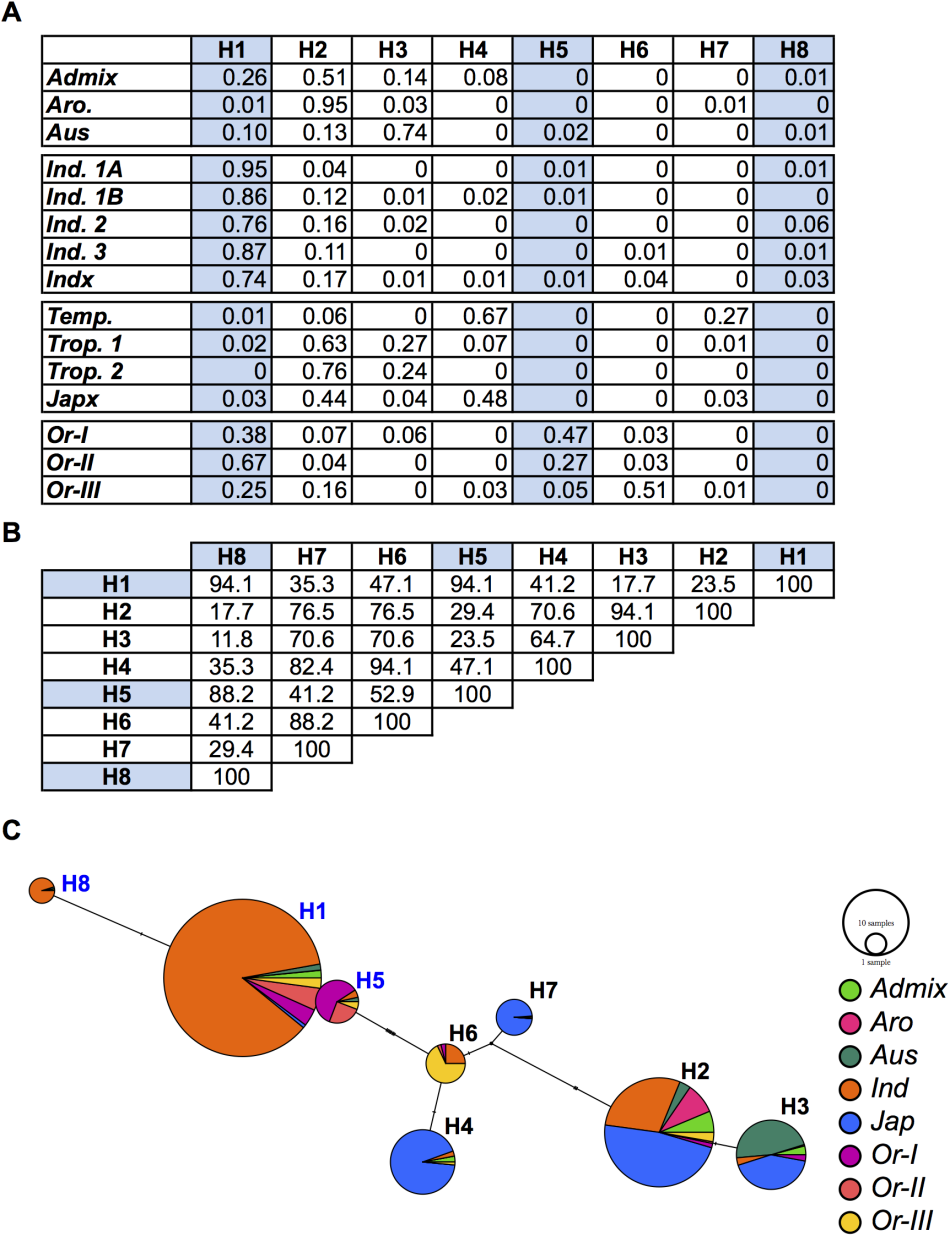
Haplotype analysis of a 37 kb region around HKT1;1 in cultivated and wild rice. (A) Haplotype frequencies in a panel of 3,024 cultivated rice accessions and 446 Oryza rufipogon accessions. (B) Pairwise comparisons of sequence similarities in between the haplotypes in A. High root Na^+^ haplotypes (i.e. those with the three non-synonymous SNPs are highlighted in blue) (C) Haplotype network for the 37 kb region surrounding *HKT1;1*. The size of each node is proportional to the number of accessions with the corresponding haplotype. Each node is separated into a pie chart, which indicates the number of accessions for each population with the haplotype. *Indica* (*Ind*) accessions are those belonging to *Ind 1A*, *Ind 1B*, *Ind 2*, *Ind 3*, and *Indx*. *Japonica* accessions are those belonging to *Temp*, *Trop 1*, *Trop 2*, and *Japx*.

A difference in allele frequencies of the three non-synonymous mutations in HKT1;1 was observed between the major subpopulations in the 32 sequenced accessions of RDP1. However, since it was difficult to examine subpopulation differentiation with this small of a sample size, the differences were explored in more depth using resequencing data from a larger diversity panel of 3,024 accessions [37]. A total of 206 SNPs spanning a ~38 Kb region around *HKT1;1* was used for haplotype analysis. In agreement with the allele frequency observed in the 32 accessions of RDP1, a clear differentiation could be observed between *indica* (*ind1A*, *ind1B*, *ind2*, *ind3* and *indx*) and *japonica* (*temp*, *trop1*, *trop2* and *japx*) subspecies in the larger diversity panel (Fig 8). Haplotypes H1, H5 and H8 harbored the three non-synonymous alleles and were found in nearly 85% of the *indica* accessions. The sequence similarity between high root Na^+^ haplotypes was very high, ranging from ~88–94% identity. Haplotypes containing high root Na^+^ alleles of *HKT1;1* were also found in the *japonica* (*temp*, *trop1*, *trop2* and *japx*), *aus* and *aromatic* subpopulations, albeit at a much lower frequency (0–3%). In contrast, haplotypes H2, H3, H4, and H7 were found predominately in the *japonica* accessions and lacked the high root Na^+^ allelic form of *HKT1;1*. Within the low root Na^+^ group, haplotypes exhibited high sequence similarity (~65–94%). Given the clear divergence between *indica* and *japonica* for *HKT1;1* haplotypes and the effects of HKT1;1 isoforms on root Na^+^ content, collectively these results strongly suggest that a significant proportion of the difference between rice subpopulations in root Na^+^ in RDP1 is due to differences in frequency of HKT1;1 variants.

Given the contrasting haplotype frequencies of high and low root Na^+^ variants of HKT1;1 between subpopulations of cultivated rice, we explored the origins of these haplotypes by examining their frequencies in a collection of 446 *Oryza rufipogon* accessions collected throughout South and Southeast Asia [38]. These accessions represent three major populations (*Or-I*, *Or-II* and *Or-III*) and provide an adequate representation of the ancestral populations of cultivated rice [38]. Two haplotypes (H1 and H5) were identified that harbored the high root Na^+^ variants of *HKT1;1*, and were found in nearly 70% of the *O*. *rufipogon* accessions. The H1 haplotype displayed the highest frequency in the *Or-II* clade and was also found in the majority of *indica* accessions, suggesting that the *indica* allele is likely derived from *Or-II*. In contrast, two haplotypes (H2 and H6) were identified with the low root Na^+^ variant and were present in only 19% of the *O*. *rufipogon* accessions. The H6 haplotype was the most frequent and present in 18% of the *O*. *rufipogon* accessions, but absent from the *japonica* cultivated rice accessions. In contrast, H2 occurred at high frequency (44%) in cultivated *japonica*, particularly the *tropical japonica* subpopulation, suggesting that H2 is potentially the ancestral haplotype for the *japonica* subspecies. Interestingly, the haplotypes found at high frequencies in the *japonica* subspecies were present at considerably lower frequencies in wild rice accessions (the highest frequency observed was 0.16), indicating that these haplotypes in *japonica* subspecies may be derived from a relatively small population of wild progenitors.

## Discussion

Salinity tolerance is a complex polygenic trait and is regulated by several physiological mechanisms [3]. Salinity reduces plant growth through osmotic effects, which are experienced shortly after the addition of Na^+^ to the external media, and ionic effects, which are experienced later in the stress as Na^+^ accumulates in the leaves to toxic levels. The ability to maintain growth in saline conditions involves a suite of physiological mechanisms including osmotic adjustment, the exclusion of sodium from leaf tissues by sequestration in the root or leaf sheath, the storage of Na^+^ into vacuoles or partitioning in tissues where the toxic effect of Na^+^ is reduced [3]. In this study, the complex polygenic nature of salinity tolerance in rice is evidenced by the large number of loci with small effects identified for shoot and root growth responses.

Although shoot Na^+^ exclusion is often used as a parameter for salt tolerance, the relationship between low shoot Na^+^ and the ability to maintain growth in saline conditions does not always hold true [39,40]. Here, no significant relationships were observed between ion traits and growth responses across all the subpopulations, suggesting that in the current experimental conditions other tolerance mechanisms besides Na^+^ exclusion may be important for salt tolerance in rice. In low to moderate salinity, the osmotic effects of high Na^+^ in the external media are likely to have a much greater impact on plant growth, compared to ionic effects [3]. During the ionic phase of salt stress, Na^+^ must accumulate to toxic levels to cause cell death and impede growth. Thus, ionic tolerance mechanisms begin to play a role much later in the stress, or when the concentration of Na^+^ in the external media is high. Other studies that have exposed diverse rice accessions to higher concentrations of NaCl and/or for longer periods have reported weak to moderate relationships between ion traits and growth responses to salinity [24,41,42]. Thus, Na^+^ exclusion may be important during more severe stress treatments than was used in the current study. The relatively moderate salt stress imposed in the current study may not be enough for Na^+^ to accumulate to toxic levels to significantly inhibit growth, and may partially explain the lack of correlation, both phenotypic and genetic, between ion traits and growth responses.

### *HKT1;1* regulates root Na^+^ content

*RNC4* harbors two Na^+^ transporter genes, *HKT1;1* and *HKT1;4*. *HKTs* are well-known components of salinity tolerance in several plant species including rice (*HKT1;5* is likely the causal gene in the *SalTol* QTL), wheat and *Arabidopsis* [25,43–51]. Although both *HKT1;1* and *HKT1;4* displayed significant differences in expression between allelic groups at *RNC4*, several key findings suggest that HKT1;1 is more important for root Na^+^ content during the early tillering stage and for the salinity level imposed in our experimental set-up. First, the genes are expressed at different developmental stages. *HKT1;1* was expressed at the highest levels in blade and leaf sheath tissues of seedlings, while *HKT1;4* showed the highest expression in culms of mature plants (Fig 4B). Second, reduced expression of *HKT1;1* in transgenic *RNAi* lines resulted in a greater sensitivity to salinity compared to *WT*, while *HKT1;4*_*RNAi*_ and *WT* plants displayed similar phenotypes under salinity (Fig 5). In a recent report, Suzuki *et al* showed that *HKT1;4* is primarily expressed in peduncles during flowering (14 week old plants) and, through *RNAi*, showed that *HKT1;4* is primarily involved in Na^+^ homeostasis only during the reproductive phase [51]. Since the current study was conducted during the early tillering stage (< 1 month old plants), it is unlikely that this gene would have an impact on salinity tolerance in this developmental window. Finally, increased expression of *HKT1;1* with the native promoter resulted in higher Na^+^ in root tissue, which is identical to the phenotype associated with *RNC4*. Together, these data suggests that *HKT1;1* is the causal gene underlying *RNC4* and contributes to root Na^+^ content during the early tillering stage.

### Variants in HKT1;1 underlie natural variation for root Na^+^ content

The differences in Na^+^ content observed between allelic groups at *RNC4* is likely due to functional differences in Na^+^ transport by *HKT1;1* alleles, with the three non-synonymous SNPs in *HKT1;1-Zh* resulting in higher Na^+^ transport activity. Na^+^ transport occurred at less negative voltages in the isoform found in accessions with high root Na^+^ compared to that isolated from accessions with low root Na^+^. During salt stress, the accumulation of Na^+^ in the apoplastic space increases HKT1;1 Na^+^ transport activity, the apparent affinity for Na^+^ of this transporter type is particularly low (Km ~ 80 mM; S12 Fig), but in the meantime, uptake of Na^+^ from the apoplast results in membrane depolarization, which reduces HKT1;1 conductance due to inward rectification property [34]. In the high root Na^+^ isoform, a higher (less negative) voltage threshold of current activation was observed, for instance in the presence of 10 mM external Na^+^ noticeable Na^+^ transport was observed between -75 and -90 mV, while in the low root Na^+^ isoforms, activation occurred at more negative voltages (Fig 6C and 6D). Thus, lower Na^+^ concentrations are required to induce Na^+^ uptake in the high root Na^+^ isoform of HKT1;1. In summary, the enhanced ability to transport Na^+^ in accessions harboring the high root Na^+^ isoform of HKT1;1 is likely due to the early activation of Na^+^ transport.

### *HKT1;1* isoforms are derived from independent populations during domestication

*Indica* varieties have long been recognized to as a source of salt tolerance, largely due to Na^+^ exclusion from leaf tissue. The most widely used QTL, *SalTol*, was identified by Lin *et al*. using a biparental population derived from the salt tolerant *indica* landrace ‘Nona Bokra’ and sensitive *japonica* variety ‘Koshihikari’ [21]. Tolerance mediated by *SalTol* is associated with the exclusion of Na^+^ from shoot tissue, through the removal of Na^+^ from the xylem and sequestration in xylem parenchyma cells in the root tissue [18,25]. While several studies have demonstrated that the *indica* subspecies harbors many varieties exhibiting high shoot Na^+^ exclusion ability, tolerant alleles in *SalTol* have only been utilized from a few *indica* landraces, and it is likely that other loci are contributing to Na^+^ exclusion in the *indica* subspecies [12,52].

In agreement with previous studies, a considerable difference among the five subpopulations was observed in root and shoot Na^+^ content and Na^+^:K^+^, with *indica* accessions generally displaying higher root Na^+^ content and Na^+^:K^+^, as well as slightly lower shoot Na^+^ and Na^+^:K^+^. The relationship between root and shoot ion traits (specifically Na^+^ and Na^+^:K^+^) differed considerably within each of the subpopulations. For instance, positive correlations were observed between tissues for Na^+^ and Na^+^:K^+^ in the *tej*, *trj* and *aus* subpopulations. However, in the *indica* and *admix* subpopulations no relationships were observed between tissues for Na^+^ and Na^+^:K^+^. The moderate positive genetic correlation observed between tissues across all accessions of RDP1 indicates that these traits may be regulated in part by common genes. However, this may be highly dependent on the subpopulation. The high frequency of the Na^+^ accumulating isoform for of HKT1;1 in the *indica* and *admix* subpopulations may “uncouple” the relationship between tissues for Na^+^ and Na^+^:K^+^.

The contrasting root Na^+^ content observed between *indica* and *japonica* accessions of RDP1 is consistent with the differences in transport activity and the frequencies of the high and low root Na^+^ isoforms of *HKT1;1*. The haplotypes of *HKT1;1* could be clearly separated into two distinct groups, corresponding to the *japonica* (H2, H3, H4 and H7) and *indica* predominate forms (H1 and H5). The high root Na^+^ haplotypes (H1, H5 and H8) were most frequent in *Oryza rufipogon*, while the low root Na^+^ haplotypes were identified in only ~31% of the *Oryza rufipogon* accessions and were nearly fixed in *japonica* accessions. The two major subspecies of *Oryza sativa* were domesticated from two geographically isolated populations of *Oryza rufipogon* [38,53]. The low diversity in *japonica* germplasm reported by several studies is consistent with a bottleneck during domestication, and suggests that the *japonica* subspecies may be derived from a relatively small founding population of *Oryza rufipogon* [38,54–56] (S15 Fig). Although the high root Na^+^ isoform was found in ~30% of the Or-III subpopulation, the founding subpopulation of *Oryza rufipogon*, it is plausible that the bottleneck experienced during domestication may have resulted in the loss of the high root Na^+^ *HKT1;1* variant from *japonica* subspecies.

### The role of *HKT1;1* in Na^+^ exclusion from shoot tissue

Like many other *HKT* members, *HKT1;1* is well-expressed in the vascular tissue of the shoot, and to a lesser extent in the root [34,48,50]. In the current study, *HKT1;1*_*RNAi*_ lines were more sensitive to salt stress, and exhibited higher shoot Na^+^ content and lower root Na^+^ content compared to *WT* plants. The expression patterns of *HKT1;1*, as well as the phenotypes exhibited by *HKT1;1*_*RNAi*_ lines are in agreement with those reported by Mäser *et al* for *AtHKT1;1* in *Arabidopsis*, suggesting that the genes may have similar physiological functions [57]. Like *HKT1;1*_*RNAi*_, *athkt1;1* knockout mutants are hypersensitive to salt stress and exhibit higher shoot Na^+^ and lower root Na^+^ [57,58]. In rice, Wang *et al* showed that *hkt1;1* knockout mutants accumulate Na^+^ in xylem sap and display a reduction in Na^+^ in phloem sap compared to *WT* [50]. These observations together with the observed accumulation of Na^+^ in shoot tissue prompted Wang *et al* to suggest that *HKT1;1* may regulate sodium exclusion from the shoot of seedlings possibly through xylem-to-phloem or parenchyma-to-xylem transfer of Na^+^ [50]. Such xylem-to-phloem transfer of Na^+^ by a HKT member has been debated in *Arabidopsis* [44,45,58]. In agreement with *hkt1;1* mutant phenotype reported by Wang *et al*, *athkt1;1* knockout mutants also exhibit higher xylem Na^+^ and lower phloem Na^+^ [44,45,50]. Although *AtHKT1;1* was initially proposed to function in the recirculation of Na^+^ from the root to the shoot (via loading of Na^+^ into the phloem in the shoots), Sunarpi *et al* later proposed that *AtHKT1;1* functions primarily in the removal of Na^+^ from the xylem sap and eventually to the phloem through symplastic diffusion [44,45]. However, a later study showed that *AtHKT1;1* was primarily involved in the retrieval of Na^+^ from the xylem in root tissue, and suggested that the function of AtHKT1;1 in shoot tissue may be dependent on the experimental conditions (discussed in [3]) [58]. For the case of *HKT1;1* in rice, further studies (outside the scope of this manuscript) are required to provide the exact mechanism for the regulation of root Na^+^ content and/or shoot Na^+^ exclusion.

Given the phenotypes exhibited by *HKT1;1*_*RNAi*_ lines, as well as the proposed function described by Wang *et al*., the absence of an association of *HKT1;1* with shoot Na^+^ or Na^+^:K^+^ is surprising [50]. If *HKT1;1* regulates retrieval of Na^+^ from the parenchyma or xylem in shoot tissues, one would expect that the high root Na^+^ allele would also have a large impact on shoot Na^+^ content. However, the concentration of Na^+^ in shoot tissue is likely more dependent on the amount of Na^+^ loaded into the xylem, and thus mechanisms which limit the delivery of Na^+^ to xylem stream would likely be more effective mechanism for shoot Na^+^ exclusion [3]. Without an effective mechanism to limit Na^+^ entry into the xylem stream in the root, very high expression of *HKT1;1*, or a highly active variant of HKT1;1 would likely be necessary to reduce shoot Na^+^ content. While the *indica* (high root Na^+^ content) variant of HKT1;1 displayed higher transport activity compared to *japonica* variant (low root Na content), it is likely that these biophysical differences are not sufficient to have an impact on shoot Na^+^ content.

Other members of the *HKT* family have been identified that are expressed in the vascular tissue of the root, and primarily function to remove Na^+^ from the xylem to limit the delivery of Na^+^ to the shoot. In rice, this function is largely achieved through the action of *HKT1;5* [25,59]. In contrast to *HKT1;1*, *HKT1;5* is mostly expressed in the root and therefore is essentially involved in xylem sap desalinization [25]. In the current study, the *SalTol* QTL that harbors *SKC1/HKT1;5* explained only a small portion of phenotypic variation for shoot Na^+^ and shoot Na^+^:K^+^ (~6%; SNP-1.11472400). Several studies have identified alleles within *SKC1/HKT1;5* that are associated with Na^+^ exclusion and salt tolerance, but it is unclear whether the effects of these alleles are as strong as those reported by Gregorio *et al*. and Bonilla *et al*. [18,20,21,23,25,60]. Given the small effect of this QTL in the current study, as well as the large number of QTL identified for shoot Na^+^ and Na^+^:K^+^, it is likely that natural variation for shoot Na^+^ and Na^+^:K^+^ involves additional genetic components in addition to *SKC1/HKT1;5*.

## Materials and methods

### Plant materials and genotyping

This study included 383 of the 421 original RDP1 accessions, as well as seven check varieties [28–30]. Accessions were obtained from the USDA-ARS Dale Bumpers Rice Research Center and purified through single seed descent before they were phenotyped. Thirty-eight accessions of RDP1 were not included because of lack of seed availability and/or poor seed quality. The set of accessions from RDP1 included 77 *indica*, 52 *aus*, 92 *temperate japonica*, 85 *tropical japonica*, 12 *groupV/aromatic*, and 56 highly admixed accessions (nine accessions were unassigned), according to the classification by Famoso *et al* [29]. A total of 365 accessions from RDP1 were genotyped using 700,000 SNPs [32]. Filtering SNPs based on minor-allele frequency (MAF > 0.05) left ~397,812 high quality SNPs (depending on the trait analyzed) [32]. Previous results indicated LD decays to 0.20 between 0.5–1.0 Mb, indicating the marker density provided by the SNP array has suitable power to detect linked causal variants of moderate to large effect QTL [32].

### Growth conditions and salt treatment

The experiment was conducted between July to Sep 2013 in a controlled green house at Lincoln, NE. Rice (*Oryza sativa*) seeds were dehusked manually and germinated in the dark for two days at 28°C in a growth cabinet (Percival Scientific). Twelve hours before transplanting seeds were exposed to light (120 μmol m^−2^ s^−1^). The green house conditions were as follows: photoperiod (16:8 day:night), temperature 25–28°C and humidity 50–80%. Seedlings were transplanted into the pots filled with Turface (Profile Products, LLC) and were grown in tap water for four days after transplanting. For the remainder of the experiment the plants were supplemented with half strength Yoshida solution (pH 5.8) [61]. Salt treatment was applied as described previously by Walia *et al*. with minor modifications [62]. Briefly, NaCl was mixed with CaCl_2_ in a 6:1 molar ratio and was added after 10 d of seedling growth. The stress treatment was started at 2.5 dS·m^-1^ which increased gradually up to 9.5 dS·m^-1^ in 4 steps over a period of four days (~2 dS·m^-1^ or 20 mM NaCl per day) to avoid any osmotic shock to the plants. The stress treatment was stabilized at 9.5 dS·m^-1^ for next two weeks. The nutrient solution pH and electrical conductivity (EC) were monitored and maintained twice daily. The pH of the nutrient solution was maintained at 5.8 using H_2_SO_4_ and KOH. Root and shoot samples were collected separately and rinsed 3 times in tap water and once in deionized water to remove excess NaCl at the completion of the experiment (14 days of 9.5 dS·m^-1^; 28 days after transplant). The samples were oven dried at 60°C for one week prior to measuring root and shoot biomass. Shoot and roots from two plants were taken for biomass measurement.

### Ion content measurement

For the large-scale screening of RDP1 dried shoot samples were ground and 200–300 mg of total material was digested with 0.1N Nitric acid (Fisher Scientific) at 70°C for 8 hrs. Root samples were weighed and digested without any grinding. Samples were diluted and cation (Na^+^ and K^+^) concentrations in the plant extract were determined with appropriate standard by dual Flame photometry (Cole Parmer, USA).

### Statistical analysis of phenotypic data

Data was combined across periods and a linear model was fit to calculate adjusted means for individual accession using the PROC GLM procedure of the Statistical Analysis System (SAS Institute, Inc.). The linear model included period (i.e., June-July or Aug-Sept), replication nested within period, tub nested within replication, accession, and accession-by-period interaction.

For the purpose of estimating variance components, a second similar linear model was fit using PROC MIXED in SAS. This time, all effects were assumed to be random effects. Broad-sense heritability (*H*^*2*^*)* on an entry-mean basis was calculated as $H^{2}=\sigma_{G}^{2}/(\sigma_{G}^{2}+\sigma_{GP}^{2}/2+\sigma_{e}^{2}/6)$ Where $\sigma_{G}^{2}$ is the variance among accessions, $\sigma_{GP}^{2}$ is the accession-by-period interaction variance, and $\sigma_{e}^{2}$ is the error variance. In this context, the divisor 2 is equal to the number of periods and the divisor 6 is equal the number of replications per period (three) multiplied by the number of periods. Broad-sense heritability provides a sense of how much of the total variation observed is due to genetic variation among accession, and indicates the power of GWAS.

### Mixed linear model for genome-wide association analysis

Marker-trait associations were tested using the linear mixed model **y = Xβ + Cγ + Zu + e** where **y** is a vector of phenotype; **β** is a vector of fixed marker effects; **γ** is a vector of principal component (PC) effects fit in order to account for population structure; **u** is a vector of polygenic effects caused by relatedness; **e** is a vector of residuals; **X** is a marker incidence matrix relating **β** to **y**; **C** is an incidence matrix relating **γ** to **y** which consists of the first four principal components (PCs) resulting from a PC analysis; **Z** is the corresponding design matrix relating **y** to **u**. It is assumed $u∼MVN(0,K\sigma_{u}^{2})$ and $e∼MVN(0,I\sigma_{e}^{2})$ where K is a standardized kinship matrix estimated using an allele-sharing matrix calculated from the SNP data. The above model was implemented using the efficient mixed-model association (EMMA) algorithm of Kang *et al* [33].

The method published by Li and Ji was used to determine a comparison-wise error rate to control the experiment-wise error rate [63]. Briefly, the correlation matrix and eigenvalue decomposition among 397,812 SNPs were calculated to determine effective number of independent tests (M_eff_). The test criteria was then adjusted using the M_eff_ with the Sidak correction below

$$\alpha_{p}=1–{\left(1\text{-}\;\alpha_{e}\right)}^{1/Meff},$$

where α_p_ is the comparison-wise error rate and α_e_ is the experiment-wise error rate [64]. An α_e =_ 0.05 was used in this study.

Analysis of variance (ANOVA) was used to estimate proportion of phenotypic variance accounted for by significant SNPs after adjusting for population structure effects. A 200 kb window was used to define groups of significant SNPs tagging the same locus. Only the most significant SNP within a 200 kb window was used to tag that locus. The percent variation explained by each significant SNPs was determined by comparing the linear models, **y = Xβ + Cγ + e**, and **y = Cγ + e**, where **β** is the SNP effect; **γ** is a vector of PCs effects to account for population structure; **X** is a vector of SNP genotypes; **C** is an incidence matrix relating **γ** to **y** which consists of the first four principal components (PCs). Therefore, the effect of each SNP is reported after accounting for the effects of population structure.

### Estimation of genetic correlation

Genetic correlations between traits were estimated with and without correcting for population structure and family relatedness. The rationale behind correcting genetic correlations for population structure is to measure the correlation independent of long-range LD between loci caused by population structure [65]. To accomplish this, a multivariate mixed model was fit as described by Wisser *et al*. including all traits as response variables; fixed experimental design effects (replication and tub nested within replication); fixed population structure effects modeled using the four PCs as above; random polygenic effects modeled using the kinship matrix as in the GWAS model described above; and random residuals assumed to independent and identically distributed [65]. Restricted maximum likelihood implemented in ASReml-R v.3.0 was used to estimate genetic and residual variances, and genetic and residual covariances among traits [66]. Estimates of genetic variances and covariances were used to calculate genetic correlations among traits. For estimation of genetic correlations uncorrected for population structure, the same methods were used except population structure and polygenic effects were not included in the mixed linear model.

### Haplotype analysis

Haplotype blocks were constructed using the four gamete method (4gamete) implemented in the software Haploview [67]. The method creates block boundaries where there is evidence of recombination between adjacent SNPs based on the presence of all four gametic types. We used a cut-off of 2%, meaning that if addition of a SNP to a block resulted in recombinant alleles at a frequency exceeding 2%, the SNP was not included in the block.

### Haplotype analysis of *HKT1;1* in cultivated rice and Oryza rufipogon

To examine the frequency of high and low root Na^+^ forms of HKT1;1 in a set of 3,023 cultivated rice and 446 *Oryza rufipogon* accessions, a set of 206 SNPs was extracted from a ~37 kb (30,700,524–30,737,580) region on chromosome 4. Sequence data for the cultivated rice was obtained from ~9 million genome-wide SNPs generated by the 3000 Rice Genomes Project (3K RGP) [37]. The 206 SNPs for *Oryza rufipogon* was obtained from riceHap3 (www.ncgr.ac.cn/ricehap3/) [38]. Since SNPs were mapped to different genome builds (IRGSP4.0 to IRGSP1.0 for 3kg and RiceHap3, respectively), the coordinates were converted by aligning a 37 kb region from IRGSP4.0 to IRGSP1.0 using BLAT [68].

Haplotype block analysis was performed using the 4Gamete rule, with a cutoff of 1% in Haploview [67]. The frequency of each haplotype within in each subpopulation was determined in R. A haplotype network for this 37 kb region was built with PopArt [69]. Nucleotide diversity (π) was determined at each position for *indica* (*ind1A*, *ind1B*, *ind2*, *ind3* and *indx*), *japonica* (*temp*, *trop 1*, *trop 2*, and *japx*) and wild rice using the "site-pi" function in VCFtools [70].

### Growth conditions and sample collection for RNA sequencing

For gene expression analysis, plants were grown in a controlled environment growth chamber. Temperatures were maintained at 28°C and 25°C in day and night respectively, relative humidity was maintained at 60% in both day and night. Lighting was maintained at 800 μmoles·m^−2^·s^−1^ using high pressure sodium lights (Phillips). Seeds preparation and salt treatment were performed as described above. Eight day (four days after transplant) old rice seedlings were subjected to 6 dS·m^-1^ for a period of 24h. Salinity stress was increased to 6 dS·m^-1^ gradually in two 3 dS·m^-1^ intervals over a period of 24h. After 24h of 6 dS·m^-1^, aerial parts of the seedlings were excised from the roots and frozen immediately in liquid nitrogen. The samples were ground with Tissuelyser II (Invitrogen) and total RNA was isolated with RNAeasy isolation kit (Qiagen) according to manufacturer’s instructions. On-column DNAse treatment was performed to remove genomic DNA contamination (Qiagen). Sequencing was performed using Illumina HiSeq 2500. Sixteen cDNA libraries were combined in each lane.

### RNA-seq mapping and quantification

After being examined using the package FastQC, short reads, obtained from Illumina 101-bp single-end RNA sequencing, were screened and trimmed using Trimmomatic to ensure each read has average quality score larger than 30 and longer than 15 bp [71,72]. The trimmed short reads were mapped against to the rice genome (*Oryza sativa* MSU Release 6.0) using TopHat (v.2.0.10), allowing up to two base mismatches per read. Reads mapped to multiple locations were discarded [73]. Numbers of reads in genes were counted by the HTSeq-count tool using gene annotations for the same version of rice genome and the “union” resolution mode was used [74].

### Variant identification

For a given genotype, all mapped RNA-seq short reads were sorted and indexed by Samtools (Version: 0.1.18) [75]. Single nucleotide polymorphisms (SNPs) and small insertions/deletions (Indels) were identified based on differences between short reads from the given genotype and the reference genome sequence with mapping quality larger than 25, read depth more than 30, but less than 500. Variations in regions of interest in the rice genome were selected with their coordinates and gene annotations.

### First strand cDNA synthesis and real-time quantitative PCR

First strand cDNA synthesis for real-time quantitative PCR (qRT-PCR) was performed using iScript Reverse Transcription Supermix (Bio-Rad Laboratories, Inc., Hercules, CA, USA) using 2 μg of total RNA. For the qPCR reaction, 3 μL of the diluted cDNA (1:20) was used in the 15 μL reaction mixture. In the qPCR reaction volume, 7.5 μL of LightCycler 480 SYBR Green I Mastermix was used (Roche Diagnostics, Indianapolis, IN, USA). The qRT-PCR was carried out using Roche LightCycle 480 II with the following parameter settings (Roche Diagnostics, Indianapolis, IN, USA): 95 ^o^C pre-incubation for 5 min, amplification was done for 40 cycles at 95 ^o^C for 20 sec and 60 ^o^C for 15 sec and extension at 72 ^o^C for 15 sec; the melting curve was set-up for 95 ^o^C, 65 ^o^C, 97 ^o^C; cooling was set-up at 40 ^o^C for 30 sec. We used two independent tissue samples, with tissue from two to three plants pooled for each sample. LOC_Os04g02820 was used as an internal reference gene, which displayed stable expression in all samples analyzed. Relative expression was determined using the delta-delta Ct method [76]. Primer sequences are provided as S9 Table.

### Transgene construction and Agrobacterium-mediated transformation

For *HKT1;1*, a 112 bp region was amplified from genomic DNA of the *japonica* rice variety ‘Kitaake’, while a 95 bp region was amplified for *HKT1;4*. The fragment from *HKT1;4* was ligated into the pENTR-D-TOPO vector, while for *HKT1;1* the fragment was inserted into pDONR221 using the BP reaction following the manufacturer’s instructions (Invitrogen). Finally, each fragment was introduced into the pANDA RNAi expression vector [77,78]. Transformation of ‘Kitaake’ calli was performed according to the methods outlined by Cheng *et al*. using the EHA-105 strain of *Agrobactrium* [79]. Calli and plants were selected on ½ strength MS media supplemented with 50 μg/ml hygromycin. The expression of *HKT1;1* and *HKT1;4* in shoot and flag leaf tissue of T1 plants, respectively, was determined using realtime PCR using the same conditions as described above. Primer sequences are provided in S9 Table.

To generate native overexpression lines for the two isoforms of HKT1;1, a ~4.3 kb fragment was amplified from ‘Nipponbare’ and ‘Zhenshan 2’. The fragments were cloned into pDONR221 vector via the BP reaction, and were subsequently cloned into a pMDC99 backbone with a NOS terminator [80]. *Agrobacterium*-mediated transformation and selection of transformants was performed as described above. T1 plants with a single insertion were selected on ½ strength MS media supplemented with 50 μg/ml hygromycin and used for phenotyping.

### Evaluation of transgenic plants

Phenotyping of transgenic plants was performed in a controlled environment growth chamber. Three independent RNAi lines (T2) for *HKT1;1* and *HKT1;4* was screened for salinity tolerance, while two independent native overexpression lines (T1 generation) were evaluated for each isoform of *HKT1;1* (*HKT1;1*_*Ni*_ and *HKT1;1*_*Zh*_). Temperatures were maintained at 28°C and 25°C in day and night respectively, relative humidity was maintained at 60% in day and night. Lighting was maintained at 800 μM using high pressure sodium lights (Phillips). Seeds were surface sterilized in a 40% bleach solution for 20 min, rinsed in sterile water and were germinated on ½ MS media supplemented with 50 ug/ml of hygromycin. *WT* seeds received the same treatment, but were grown on ½ MS. The seeds were germinated for 24h in complete darkness then were transferred to a growth cabinet (Percival Scientific) and grown for four days at 28°C in 16/8h light (120 μmol m^−2^ s^−1^). Seedlings were transplanted into the pots filled with Turface (Profile Products, LLC) and were grown in tap water for four days after transplanting. For the remainder of the experiment the plants were supplemented with half strength Yoshida solution (pH 5.8) [61]. Eight days after transplanting a gradual salt stress was applied in three 3 dS·m^-1^ intervals over a period of 24h. The final 9 dS·m^-1^ salt level was maintained for two weeks. Sample collection was performed as described above.

### Electrophysiology

To generate constructs for assessing Na^+^ transport activities in *Xenopus laevis* oocytes, *HKT1;1* was amplified from cDNA from ‘Nipponbare’ and ‘Zhenshan 2’, which are representative accessions for the high and low root Na^+^ groups at *RNC4*, respectively, and ligated into the pGEM-Xho vector [81]. The pGEM-Xho contains the T7 promoter and 5′- and 3′-untranslated regions of the *Xenopus β-globin* gene, which enhances expression in *Xenopus* oocytes. For N-terminal GFP::HKT1;1 fusion constructs, *HKT1;1* was amplified from cDNA from ‘Nipponbare’ and ‘Zhenshan 2’ and cloned into pGWB6 using the Gateway LR reaction. GFP::HKT1;1 was then amplified from each construct using primers with SpeI and SalI restriction sites, and introduced into the pGEM-Xho vector [82].

Capped and polyadenylated RNA were obtained from linearized vector by *in vitro* transcription, using the mMESSAGE mMACHINE T7 kit (Ambion, USA). Oocytes isolated as previously described were injected with 50 ng of *HKT1;1-Ni* or *HKT1;1-Zh* cRNA (equivalent amount of transporter cRNA in GFP-tagged form) in 50 nL of RNase-free water, or with 50 nL of RNase-free water (for control oocytes), and then kept for 24 to 48 h at 19°C in ND96 medium (96 mM NaCl, 2 mM KCl, 1.8 mM CaCl_2_, 1 mM MgCl_2_, 2.5 mM sodium pyruvate, and 5 mM HEPES/NaOH, pH 7.4) supplemented with 0.5 mg·L^–1^ of gentamicin, until experiments [81]. Whole oocyte currents and membrane potential were recorded using the two-electrode voltage-clamp technique with a GeneClamp 500B amplifier (Axon Instruments, USA) 1 to 2 days after cRNA injection. Voltage-pulse protocols, data acquisition and analysis were performed using pClamp9 software (Axon Instruments). Correction was made for voltage drop through the series resistance of the bath and the reference electrode using two external electrodes connected to a bath probe (VG-2A x100 Virtual-ground bath clamp; Axon Instruments). Electrodes were filled with 3 M KCl. The oocytes were continuously perfused during the voltage-clamp experiment with bath solutions containing varying concentrations of monovalent cations (as glutamate or chloride salts) in a background of 6 mM MgCl_2_, 1.8 mM CaCl_2_, and 10 mM MES-1,3-bis[tris(hydroxymethyl) methylamino]propane, pH 5.5. The chloride concentration was constant in each set of solutions. D-Mannitol was added when necessary to adjust the osmolarity, which was set to 220–240 mosM in each set of solutions. Voltage-clamp protocol consisted in successive steps of membrane voltage application from -165 to +15 mV in +15 mV increments during 0.5 s, each step beginning with 0.15 s and ending with 0.3 s at the resting potential of the oocyte membrane in the tested bath solution. Mean currents recorded in water-injected control oocytes from the same batch and in the same ionic conditions as HKT-expressing ones were subtracted from those recorded in HKT-expressing oocytes in order to extract HKT-mediated currents from total oocyte currents. HKT1;1-Ni and -Zh current–voltage (I–V) relationships were constructed with transporter extracted currents. The activation potential of HKT currents was estimated as the lowest voltage at which the current in HKT-expressing oocytes reached twice that in control oocytes.

Confocal observations were made on dark poles of oocytes of similar sizes on a Leica SP8 microscope, using a 20x/0.7dry objective. GFP was excited with a 488 nm laser, and spectral acquisitions of emitted fluorescent light were performed between 495 and 645 nm using a bandwidth of 5 nm, to assert GFP specificity. For each oocyte, mean fluorescence intensity at the membrane was determined from at least 2 optical sections, analyzing 3 ROIs per section using ImageJ (https://imagej.nih.gov/ij/) software.

## Supporting information

S1 Fig GWA of root K^+^ content in salt conditions. Genome-wide association (GWA) was performed using a mixed model that accounted for population structure and relatedness between accessions of RDP1 using 365 accessions of RDP1 and 397,812 SNPs. For each trait the least squares mean was used as the dependent variable. The red horizontal line indicates a statistical significance threshold of p < 10^−5^, and was determined using the M_eff_ method with an experiment-wise error rate of 0.05 [63].
(TIF)

S2 Fig GWA of shoot ion traits in salt conditions. Genome-wide association (GWA) was performed for (A) Na^+^ content, (B) K^+^ content, and (C) Na^+^:K^+^ using a mixed model that accounted for population structure and relatedness between accessions of RDP1 using 365 accessions of RDP1 and 397,812 SNPs. For each trait the least squares mean was used as the dependent variable. The red horizontal line indicates a statistical significance threshold of p < 10^−5^, and was determined using the M_eff_ method with an experiment-wise error rate of 0.05 [63].
(TIF)

S3 Fig GWA of shoot biomass traits. (A) Shoot biomass in control conditions; (B) shoot biomass in saline conditions; (C) shoot biomass response. Genome-wide association (GWA) was performed using a mixed model that accounted for population structure and relatedness between accessions of RDP1 using 365 accessions of RDP1 and 397,812 SNPs. The red horizontal line indicates a statistical significance threshold of *p* < 10^−5^, and was determined using the M_eff_ method with an experiment-wise error rate of 0.05 [63]. For each trait the least squares mean determined for accession within each condition (i.e. salt or control). The ratio of biomass in salt to control was used to identify loci associated with the effect of saline treatment on the growth.
(TIF)

S4 Fig GWA of root biomass traits. (A) Root biomass in control conditions; (B) root biomass in saline conditions; (C) root biomass response. Genome-wide association (GWA) was performed using a mixed model that accounted for population structure and relatedness between accessions of RDP1 using 365 accessions of RDP1 and 397,812 SNPs. The red horizontal line indicates a statistical significance threshold of *p* < 10^−5^, and was determined using the M_eff_ method with an experiment-wise error rate of 0.05 [63]. For each trait the least squares mean determined for accession within each condition (i.e. salt or control). The ratio of biomass in salt to control was used to identify loci associated with the effect of saline treatment on the growth.
(TIF)

S5 Fig Haplotype blocks for the region spanning 30.48–30.6 Mb on chromosome 4. The heavy black line indicates the boundaries of individual blocks, which were determined using the 4Gamete rule in Haploview with a recombination threshold of > 2%. The track above the heatmap indicates the position of the SNP within the defined region. Each cell represents the strength of LD between the two SNPs with darker red indicating high LD. Haplotype block analysis was performed for the entire region spanning RNC4, but to visual aesthetics the figures are presented separately.
(TIF)

S6 Fig Haplotype blocks for the region spanning 30.6–30.7 Mb on chromosome 4. The heavy black line indicates the boundaries of individual blocks, which were determined using the 4Gamete rule in Haploview with a recombination threshold of > 2%. The track above the heatmap indicates the position of the SNP within the defined region. Each cell represents the strength of LD between the two SNPs with darker red indicating high LD. Haplotype block analysis was performed for the entire region spanning RNC4, but to visual aesthetics the figures are presented separately.
(TIF)

S7 Fig Haplotype blocks for the region spanning 30.8–30.9 Mb on chromosome 4. The heavy black line indicates the boundaries of individual blocks, which were determined using the 4Gamete rule in Haploview with a recombination threshold of > 2%. The track above the heatmap indicates the position of the SNP within the defined region. Each cell represents the strength of LD between the two SNPs with darker red indicating high LD. Haplotype block analysis was performed for the entire region spanning RNC4, but to visual aesthetics the figures are presented separately.
(TIF)

S8 Fig Haplotype blocks for the region spanning 30.9–31.06 Mb on chromosome 4. The heavy black line indicates the boundaries of individual blocks, which were determined using the 4Gamete rule in Haploview with a recombination threshold of > 2%. The track above the heatmap indicates the position of the SNP within the defined region. Each cell represents the strength of LD between the two SNPs with darker red indicating high LD. Haplotype block analysis was performed for the entire region spanning RNC4, but to visual aesthetics the figures are presented separately.
(TIF)

S9 Fig Expression of *HKT1;1* and *HKT1;4* in root and shoot tissue. RNA sequencing of root and shoot tissue was performed with two accessions of RDP1 at 10 days after transplanting (12 day old plants) when the first tiller was visibly emerging. The expression levels of *HKT1;1* and *HKT1;4* are expressed as reads per kilobase per million mapped reads (RPKM). RPKM was determined using EdgeR [83] and MSUv7 annotation was used to determine the length of each gene.
(TIF)

S10 Fig **Expression of *HKT1;1* (A) and *HKT1;4* (B) in RNAi plants.** For *HKT1;1*, gene expression was quantified in whole shoot tissue in four-day-old T1 plants using real-time PCR, while for *HKT1;4* expression was quantified from flag leaf tissue at anthesis. All expression is expressed relative to developmentally identical ‘Kitaake’ plants using the using the delta-delta Ct method, with LOC_Os04g02820 as an internal reference gene [76]. Error bars represent standard error of the mean where n = 4 biological replicates.
(TIF)

S11 Fig Shoot and root growth in RNAi lines. T2 RNAi lines of HKT1;1 (A,B) and HKT1;4 (C,D) were exposed to 14d of 9 dS m^-1^. Biomass was significantly reduced by salt treatment in all lines (*p* < 0.05). Error bars represent standard error of the mean where *n* = 12–20 plants. Kitaake was used as a WT control in all experiments.
(TIF)

S12 Fig Position and conservation of non-synonymous variants in HKT1;1.2. (A) Secondary structure of OsHKT1;1 polypeptide showing the position of AA changes, as exemplified between ‘Nipponbare’ and ‘Zhenshan 2’ variants. (B) Protein alignment of OsHKT1;1.2 and HKT1;1 homologs from various species. Black triangles indicate position of three non-synonymous mutations present in HKT1;1-Zh.
(TIF)

S13 Fig HKT1;1-Ni and HKT1;1-Zh display similar affinity for Na^+^. (A, B) Current voltage (I-V) relationships determined in solutions containing 3 to 100 mM Na^+^-glutamate in HKT1;1-Ni (A) or HKT1;1-Zh (B) expressing oocytes from the same batch. (C) HKT1;1-Ni or -Zh inward conductance plotted versus external Na^+^ concentration. Whole oocyte HKT1;1 inward conductances, were determined from I-V data shown in (A) and (B) at membrane voltages from -120 to -150 mV. HKT1;1-Ni and–Zh conductances were not significantly different as determined using Student’s t test (*p*> 0.05). The concentration at which half saturation of the mean conductance occurred (apparent Km) and the maximal conductance were determined with hyperbolic fits (Michaelis-Menten equation): K_m_ = 72 mM and G_max_ = 0.38 mS for HKT1;1-Ni, K_m_ = 86 mM and Gmax = 0.51 mS for HKT1;1-Zh. Data in (A-C) are means ± SE.
(TIF)

S14 Fig HKT1;1-Ni and HKT1;1-Zh display high selectivity for Na^+^ against other monovalent cations. **(A-D)** Oocytes expressing HKT1;1-Ni (A, C) or HKT1;1-Zh (B, D) were successively bathed with solutions containing 50 mmoles•l^-1^ of different monovalent cations (Na^+^, K^+^, Li^+^, Rb^+^, or Cs^+^, as chloride salts)(A, B) or different combinations of Na^+^ and K^+^ concentrations (as glutamate salts)(C, D). Shown I-V relationships were drawn using normalized currents (to those recorded in each oocyte at -165 mV in 50 mM Na^+^ in (A) and (B), and in 10 mM Na^+^ and 0.3 mM K^+^ in (C) and (D)), in order to suppress small differences in expression level between oocytes. Data are means ± SE.
(TIF)

S15 Fig Comparisons of nucleotide diversity between cultivated and wild rice in the region surrounding *HKT1;1*. The three non-synonymous SNPs are highlighted in red. The y-axis represents the ratio of the nucleotide diversity (pi) in *Oryza rufipogon* to *Oryza sativa japonica* or *Oryza sativa indica*.
(TIF)

S1 Table Broad-sense heritability (H^2^) estimates for the ten traits recorded. Broad sense-heritability was determined on an entry-mean basis using all 390 accessions (383 from RDP1 and seven check varieties). Period indicates the time of year the experiment was conducted (i.e. June-July or Aug-Sept), and genotype indicates the accession
(XLSX)

S2 Table Genetic correlation analysis of root and shoot ion traits. Restricted maximum likelihood implemented in ASReml-R v.3.0 was used to estimate genetic variances and covariances among traits, which were used to calculate genetic correlations among traits [66]. Here, population structure and polygenic effects were not included in the mixed linear model
(XLSX)

S3 Table Pearson correlation of eight phenotypic traits performed across all subpopulations in RDP1 (*n* = 383). SB and RB indicate the ratio of biomass in salt to control for shoots and roots, respectively. Highlighted cells indicate a statistically significant relationship (*p* < 0.05). The number within each cell indicates Pearson’s correlation coefficient.
(XLSX)

S4 Table Pearson correlation of eight phenotypic traits performed within the *admix* subpopulation (*n* = 53). SB and RB indicate the ratio of biomass in salt to control for shoots and roots, respectively. Highlighted cells indicate a statistically significant relationship (*p* < 0.05). The number within each cell indicates Pearson’s correlation coefficient.
(XLSX)

S5 Table Pearson correlation of eight phenotypic traits performed within the *tropical japonica* subpopulation (*n* = 84). SB and RB indicate the ratio of biomass in salt to control for shoots and roots, respectively. Highlighted cells indicate a statistically significant relationship (*p* < 0.05). The number within each cell indicates Pearson’s correlation coefficient.
(XLSX)

S6 Table Pearson correlation of eight phenotypic traits performed within the *aus* subpopulation (*n* = 52). SB and RB indicate the ratio of biomass in salt to control for shoots and roots, respectively. Highlighted cells indicate a statistically significant relationship (*p* < 0.05). The number within each cell indicates Pearson’s correlation coefficient.
(XLSX)

S7 Table Pearson correlation of eight phenotypic traits in the *temperate japonica* subpopulation (*n* = 91). SB and RB indicate the ratio of biomass in salt to control for shoots and roots, respectively. Highlighted cells indicate a statistically significant relationship (*p* < 0.05). The number within each cell indicates Pearson’s correlation coefficient.
(XLSX)

S8 Table Pearson correlation of eight phenotypic traits performed within the *indica* subpopulation (*n* = 77). SB and RB indicate the ratio of biomass in salt to control for shoots and roots, respectively. Highlighted cells indicate a statistically significant relationship (*p* < 0.05). The number within each cell indicates Pearson’s correlation coefficient.
(XLSX)

S9 Table List of primer sequences used for gene expression analysis, cloning and SNP confirmation. (XLSX)

S1 File Least-squares means for the ten phenotypic traits recorded for 390 accessions. (XLSX)

S2 File QTL locations for the eight phenotypic traits and the percent variation explained by the most significant SNP for each QTL. (XLSX)

S3 File Transcripts within *RNC4* displaying differences in expression between allelic groups at SNP-4-30535352 (XLSX)

S4 File High-confidence genetic variants (at least 10x coverage) found in the ORFs of genes within *RNC4*. (XLSX)
