# Supplementary figures and images for: Allelic variants of OsHKT1;1 underlie the divergence between indica and japonica subspecies of rice (Oryza sativa) for root sodium content
Source: PLoS Genet. 2017 Jun 5;13(6):e1006823. doi: 10.1371/journal.pgen.1006823 (PMC5476289; doi:10.1371/journal.pgen.1006823)

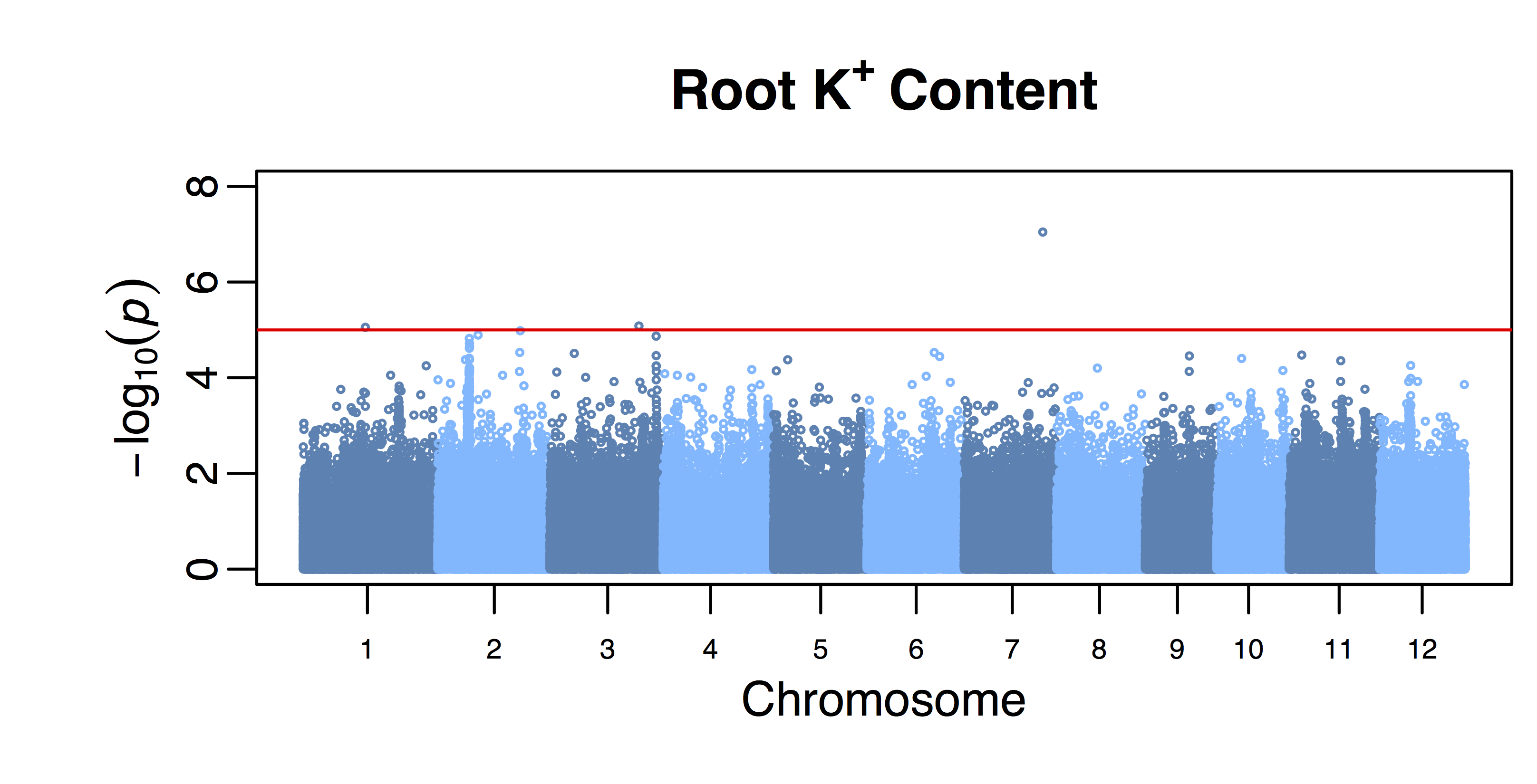

Supplement: S1 Fig — Genome-wide association (GWA) was performed using a mixed model that accounted for population structure and relatedness between accessions of RDP1 using 365 accessions of RDP1 and 397,812 SNPs. For each trait the least squares mean was used as the dependent variable. The red horizontal line indicates a statistical significance threshold of p < 10−5, and was determined using the Meff method with an experiment-wise error rate of 0.05 [63]. (TIF) [file pgen.1006823.s001.tif]

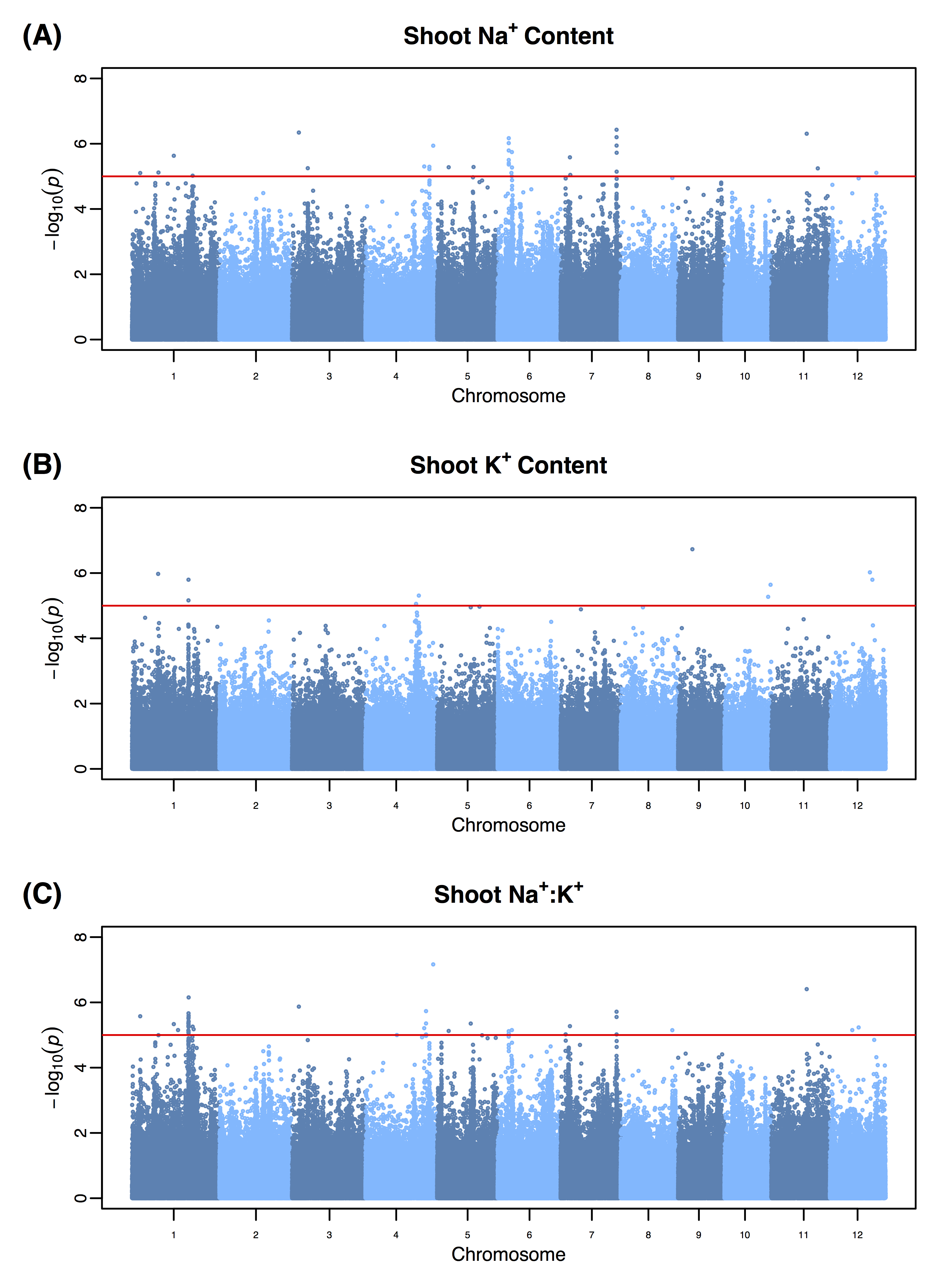

Supplement: S2 Fig — Genome-wide association (GWA) was performed for (A) Na+ content, (B) K+ content, and (C) Na+:K+ using a mixed model that accounted for population structure and relatedness between accessions of RDP1 using 365 accessions of RDP1 and 397,812 SNPs. For each trait the least squares mean was used as the dependent variable. The red horizontal line indicates a statistical significance threshold of p < 10−5, and was determined using the Meff method with an experiment-wise error rate of 0.05 [63]. (TIF) [file pgen.1006823.s002.tif]

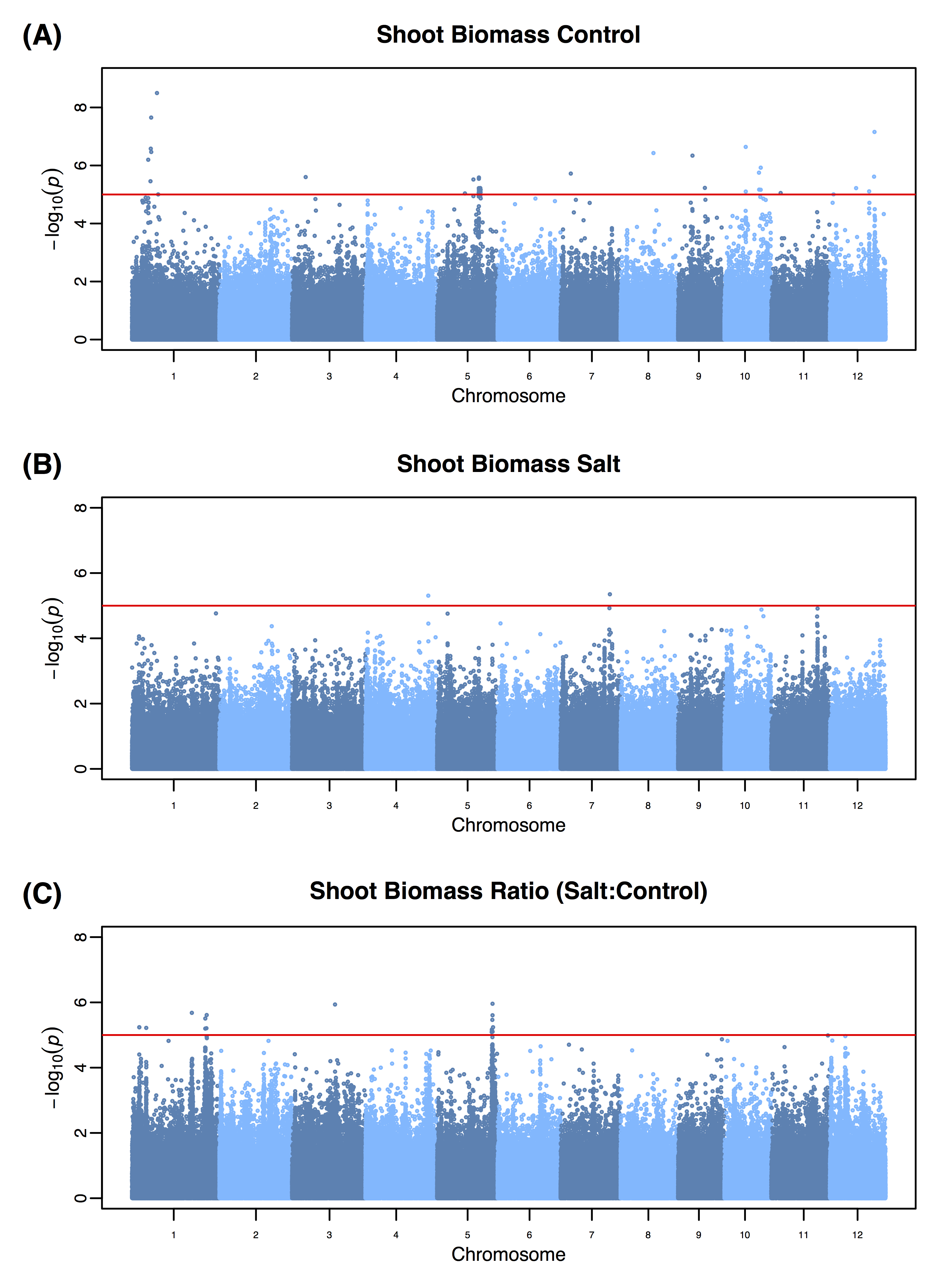

Supplement: S3 Fig — (A) Shoot biomass in control conditions; (B) shoot biomass in saline conditions; (C) shoot biomass response. Genome-wide association (GWA) was performed using a mixed model that accounted for population structure and relatedness between accessions of RDP1 using 365 accessions of RDP1 and 397,812 SNPs. The red horizontal line indicates a statistical significance threshold of p < 10−5, and was determined using the Meff method with an experiment-wise error rate of 0.05 [63]. For each trait the least squares mean determined for accession within each condition (i.e. salt or control). The ratio of biomass in salt to control was used to identify loci associated with the effect of saline treatment on the growth. (TIF) [file pgen.1006823.s003.tif]

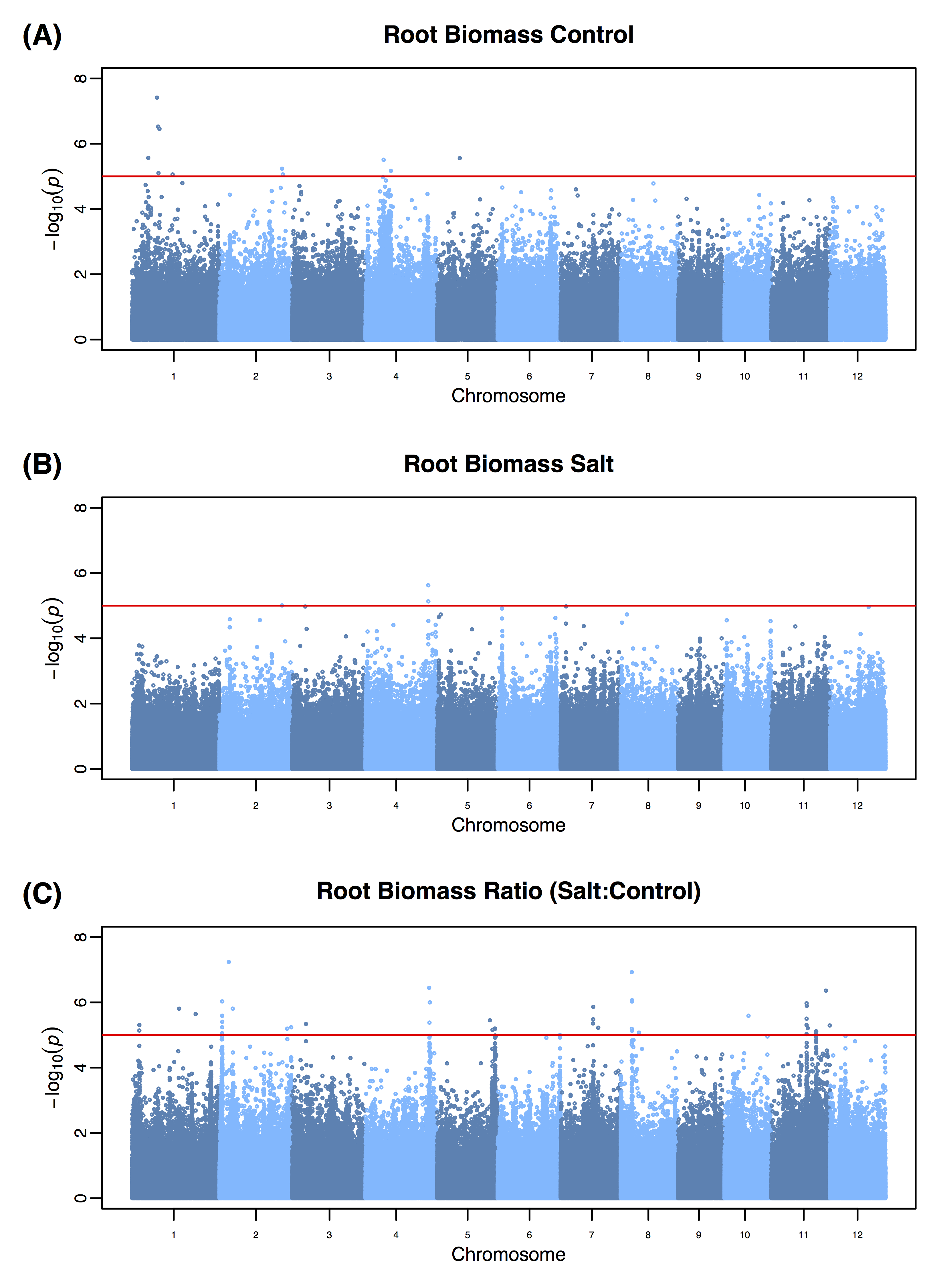

Supplement: S4 Fig — (A) Root biomass in control conditions; (B) root biomass in saline conditions; (C) root biomass response. Genome-wide association (GWA) was performed using a mixed model that accounted for population structure and relatedness between accessions of RDP1 using 365 accessions of RDP1 and 397,812 SNPs. The red horizontal line indicates a statistical significance threshold of p < 10−5, and was determined using the Meff method with an experiment-wise error rate of 0.05 [63]. For each trait the least squares mean determined for accession within each condition (i.e. salt or control). The ratio of biomass in salt to control was used to identify loci associated with the effect of saline treatment on the growth. (TIF) [file pgen.1006823.s004.tif]

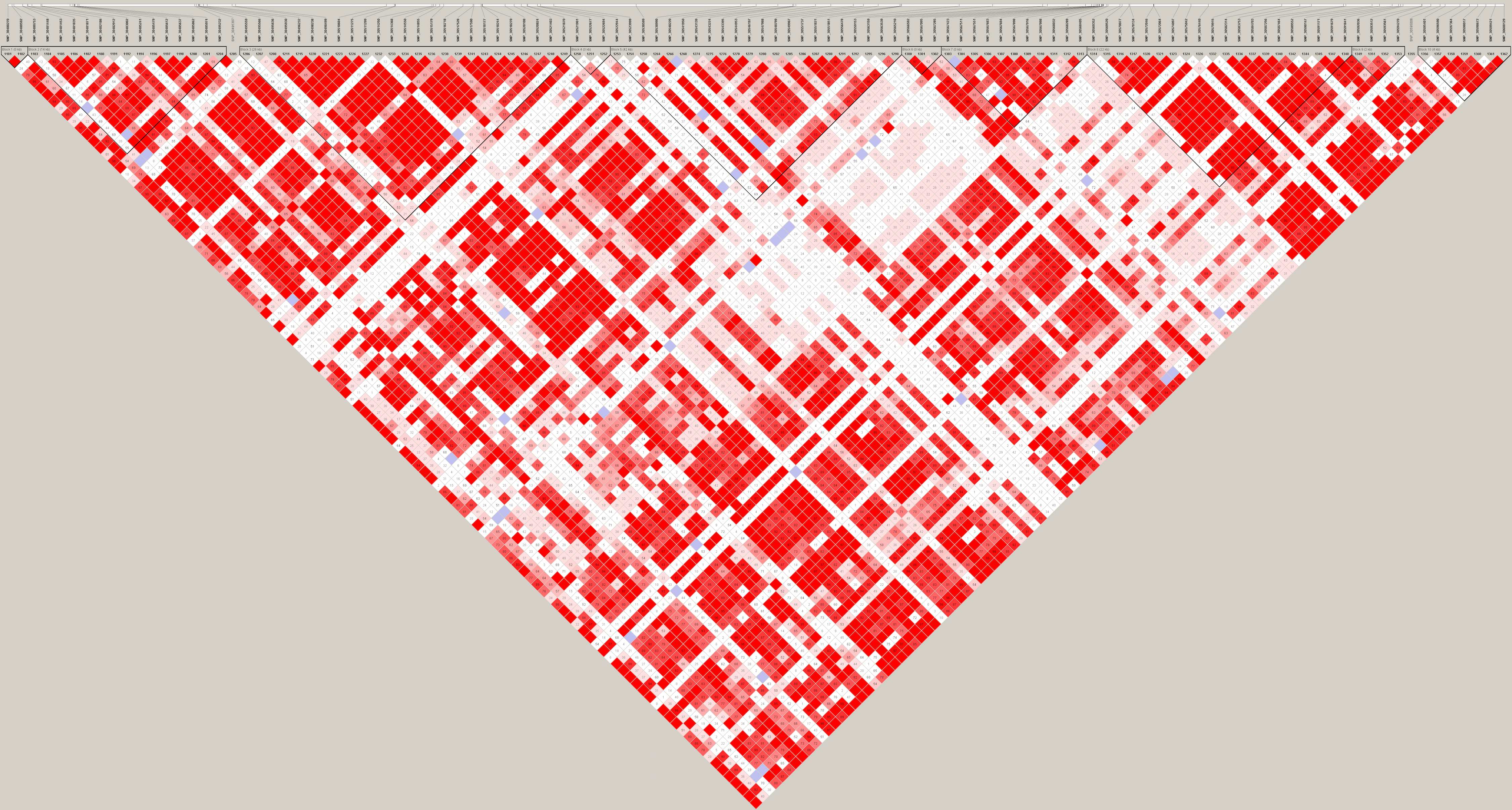

Supplement: S5 Fig — The heavy black line indicates the boundaries of individual blocks, which were determined using the 4Gamete rule in Haploview with a recombination threshold of > 2%. The track above the heatmap indicates the position of the SNP within the defined region. Each cell represents the strength of LD between the two SNPs with darker red indicating high LD. Haplotype block analysis was performed for the entire region spanning RNC4, but to visual aesthetics the figures are presented separately. (TIF) [file pgen.1006823.s005.tif]

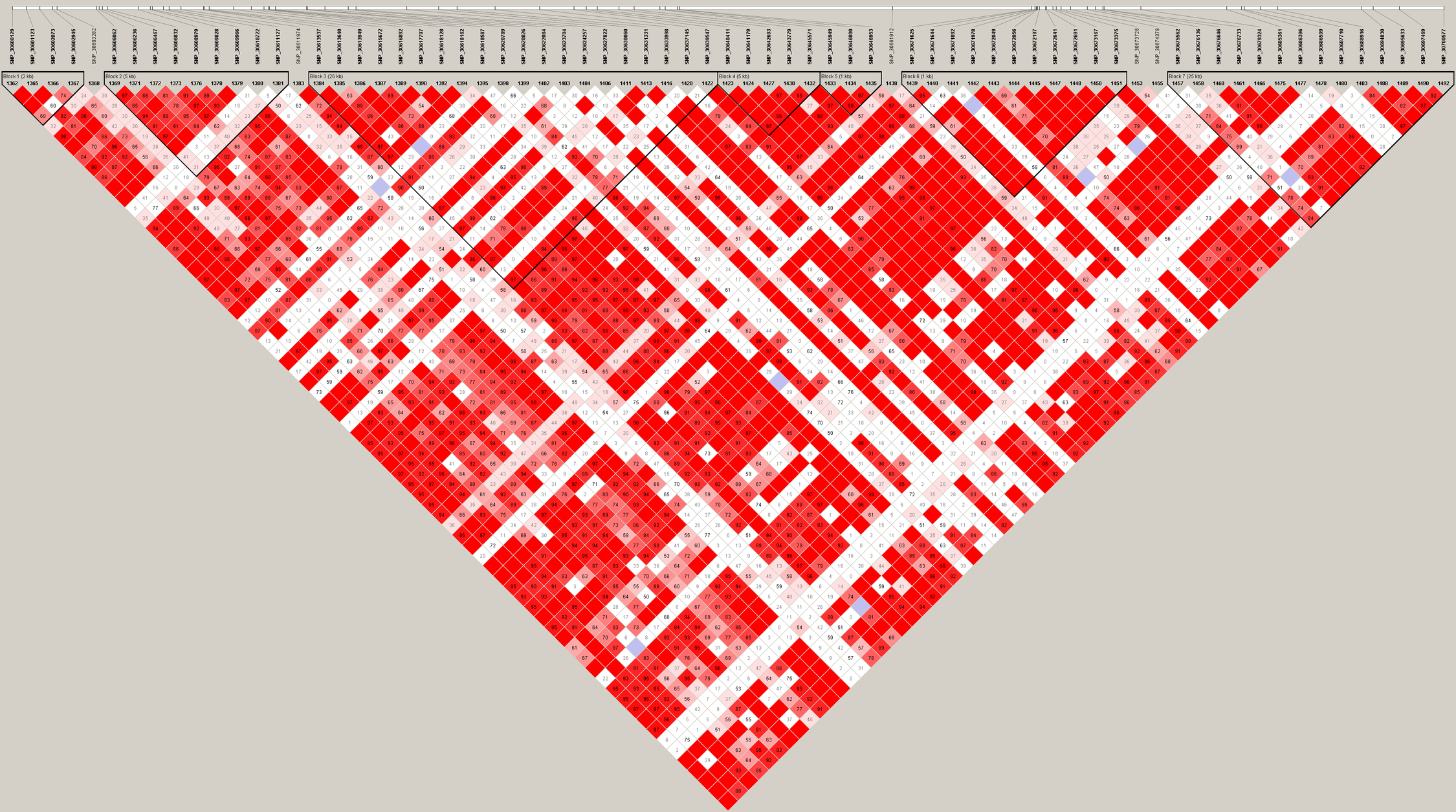

Supplement: S6 Fig — The heavy black line indicates the boundaries of individual blocks, which were determined using the 4Gamete rule in Haploview with a recombination threshold of > 2%. The track above the heatmap indicates the position of the SNP within the defined region. Each cell represents the strength of LD between the two SNPs with darker red indicating high LD. Haplotype block analysis was performed for the entire region spanning RNC4, but to visual aesthetics the figures are presented separately. (TIF) [file pgen.1006823.s006.tif]

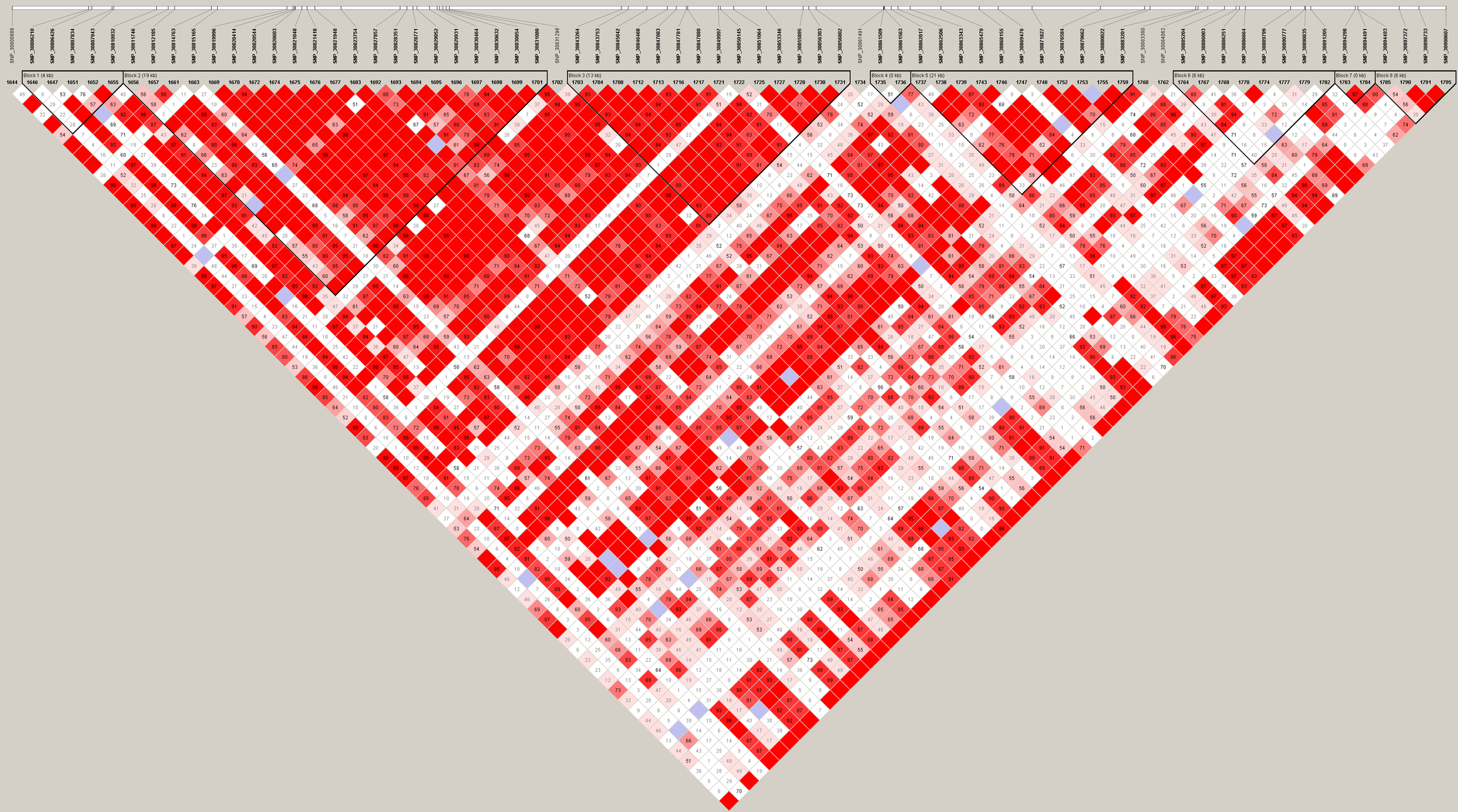

Supplement: S7 Fig — The heavy black line indicates the boundaries of individual blocks, which were determined using the 4Gamete rule in Haploview with a recombination threshold of > 2%. The track above the heatmap indicates the position of the SNP within the defined region. Each cell represents the strength of LD between the two SNPs with darker red indicating high LD. Haplotype block analysis was performed for the entire region spanning RNC4, but to visual aesthetics the figures are presented separately. (TIF) [file pgen.1006823.s007.tif]

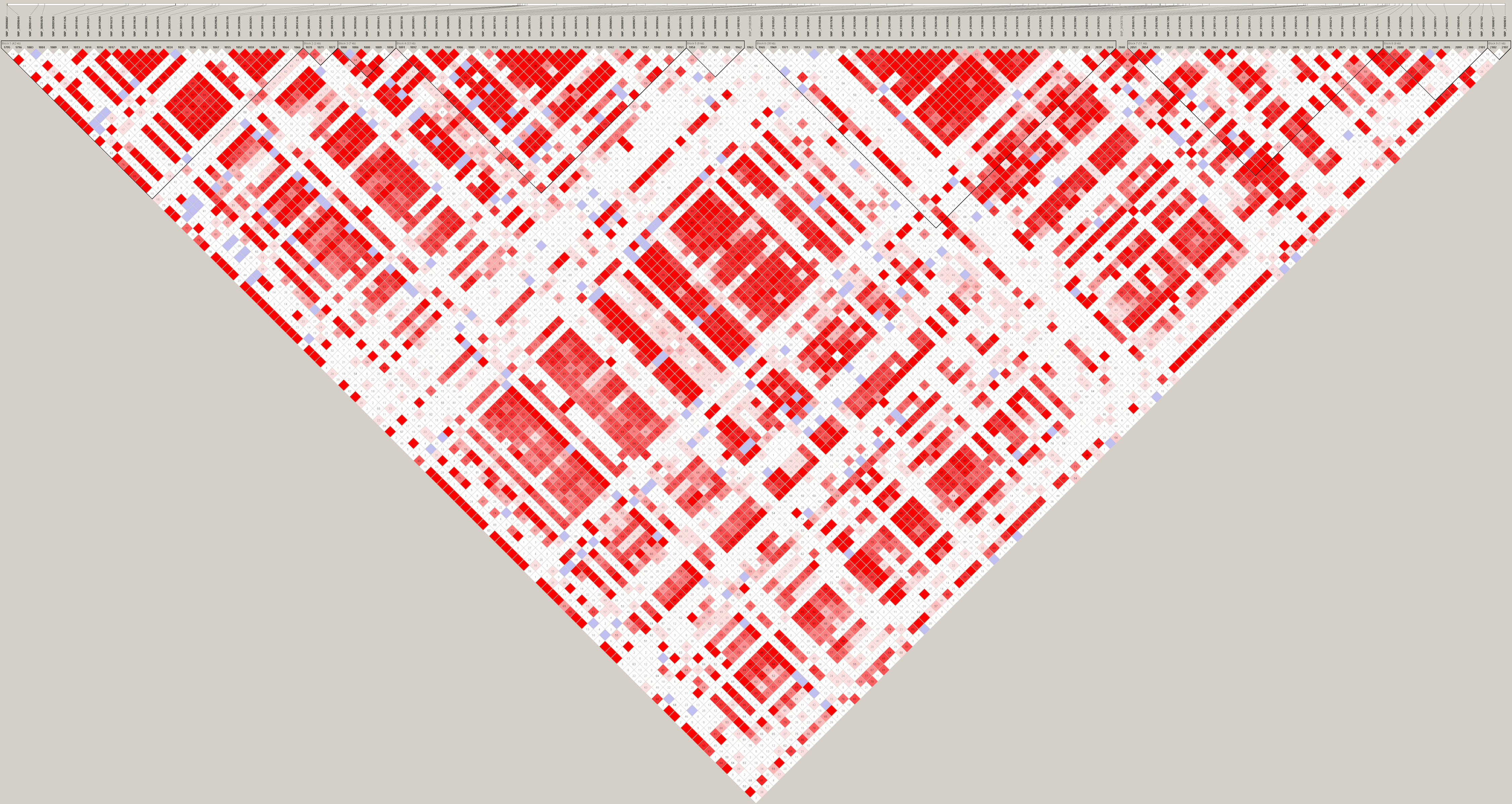

Supplement: S8 Fig — The heavy black line indicates the boundaries of individual blocks, which were determined using the 4Gamete rule in Haploview with a recombination threshold of > 2%. The track above the heatmap indicates the position of the SNP within the defined region. Each cell represents the strength of LD between the two SNPs with darker red indicating high LD. Haplotype block analysis was performed for the entire region spanning RNC4, but to visual aesthetics the figures are presented separately. (TIF) [file pgen.1006823.s008.tif]

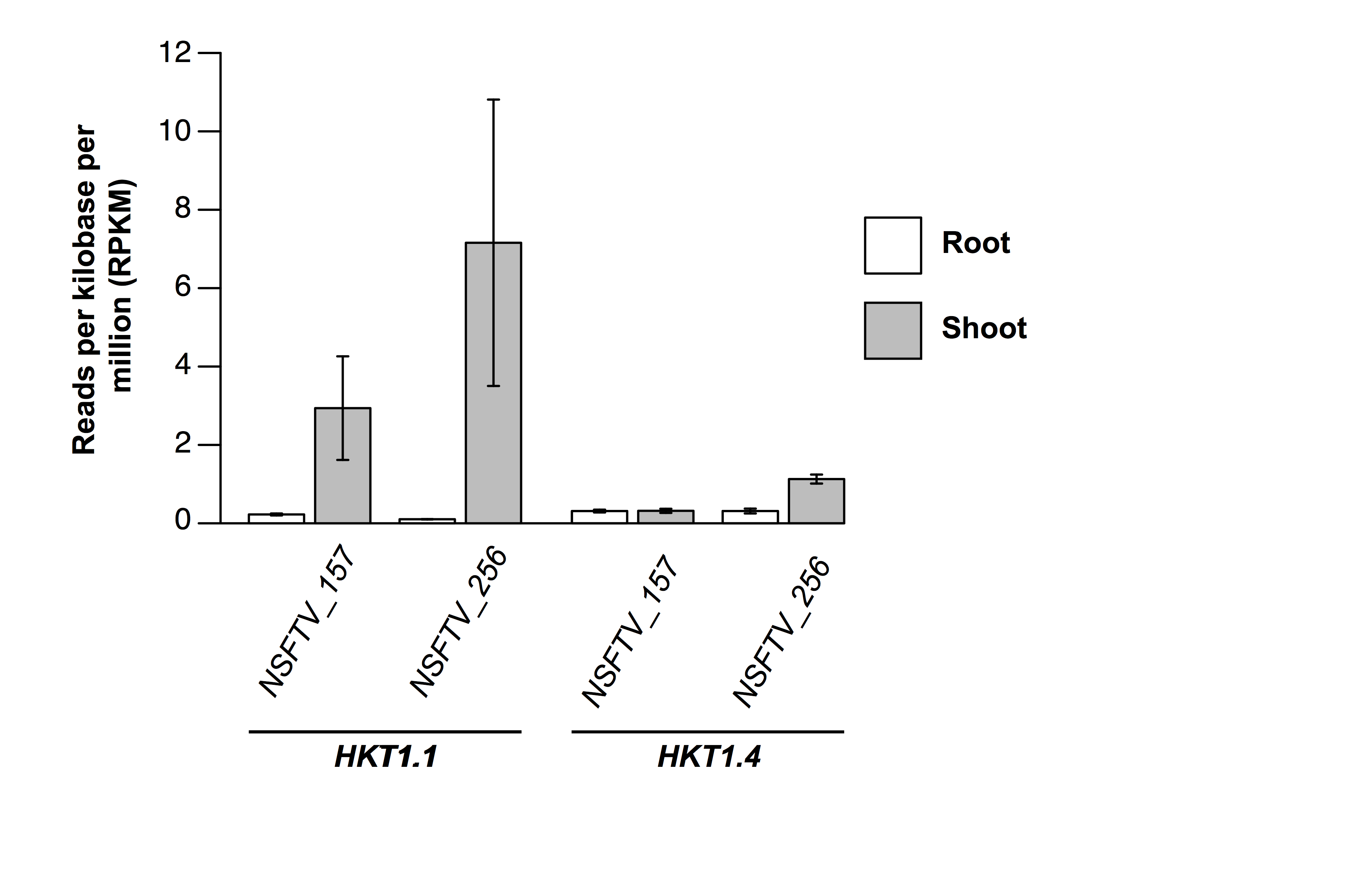

Supplement: S9 Fig — RNA sequencing of root and shoot tissue was performed with two accessions of RDP1 at 10 days after transplanting (12 day old plants) when the first tiller was visibly emerging. The expression levels of HKT1;1 and HKT1;4 are expressed as reads per kilobase per million mapped reads (RPKM). RPKM was determined using EdgeR [83] and MSUv7 annotation was used to determine the length of each gene. (TIF) [file pgen.1006823.s009.tif]

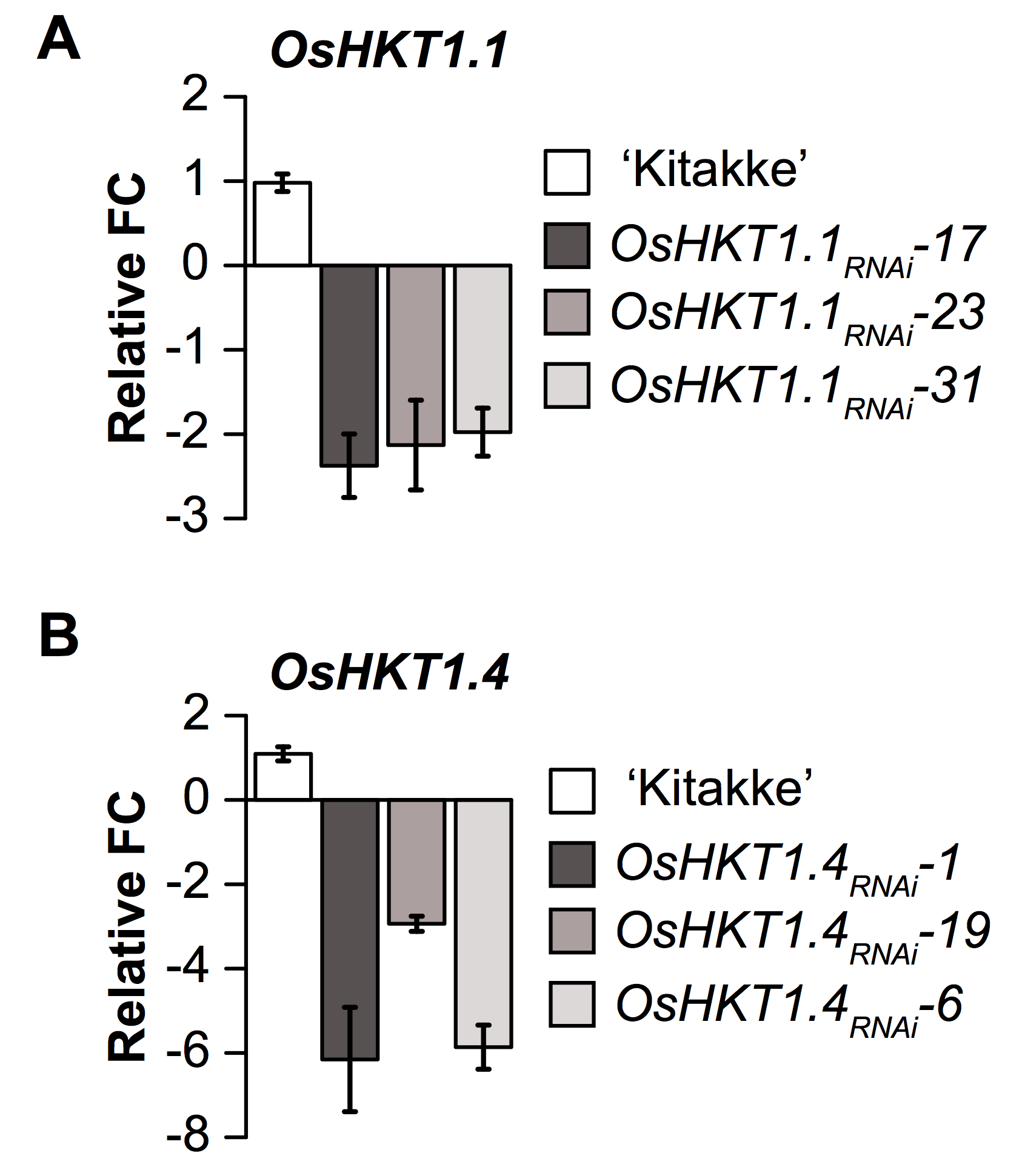

Supplement: S10 Fig — Expression of HKT1;1 (A) and HKT1;4 (B) in RNAi plants. For HKT1;1, gene expression was quantified in whole shoot tissue in four-day-old T1 plants using real-time PCR, while for HKT1;4 expression was quantified from flag leaf tissue at anthesis. All expression is expressed relative to developmentally identical ‘Kitaake’ plants using the using the delta-delta Ct method, with LOC_Os04g02820 as an internal reference gene [76]. Error bars represent standard error of the mean where n = 4 biological replicates. (TIF) [file pgen.1006823.s010.tif]

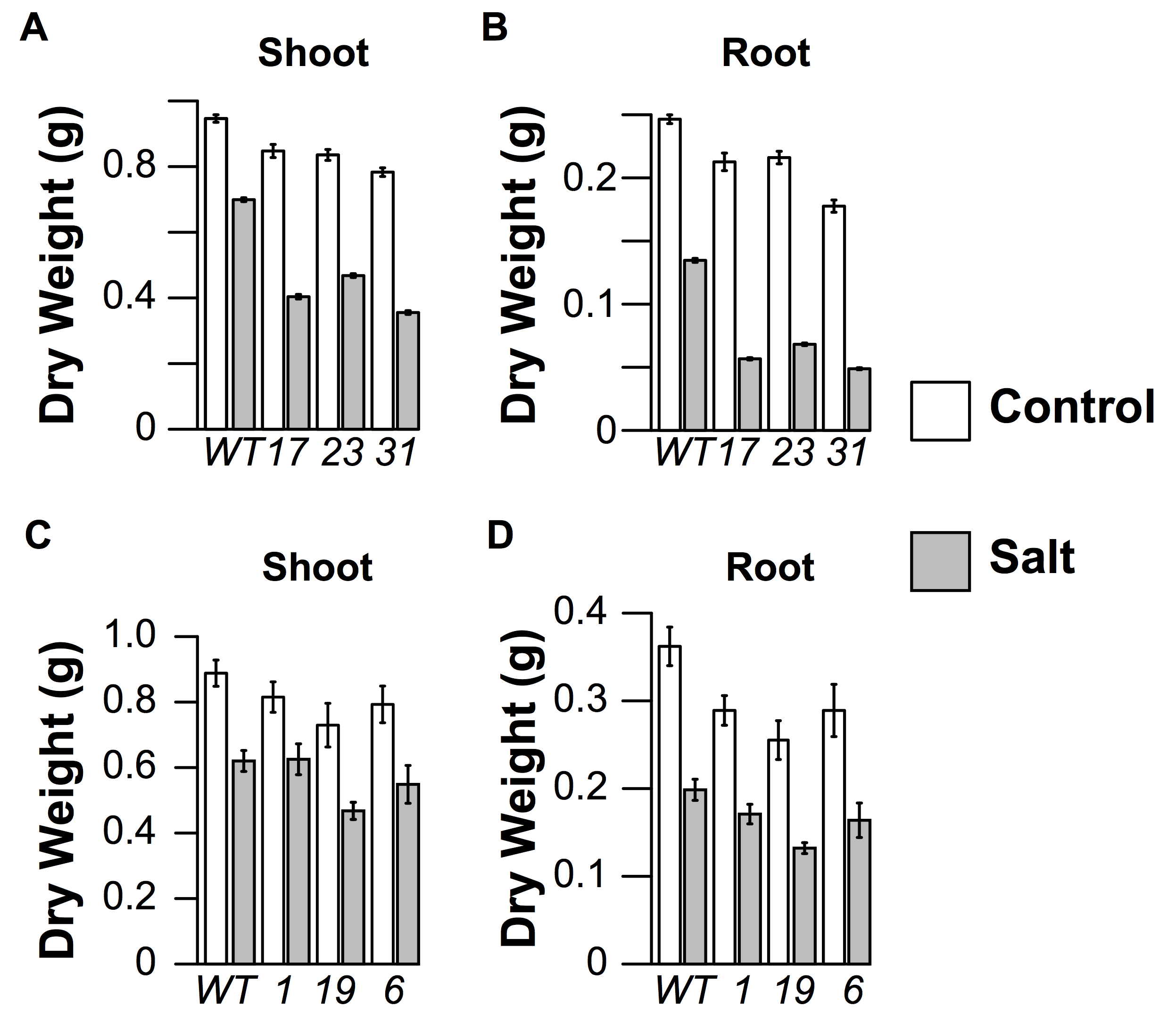

Supplement: S11 Fig — T2 RNAi lines of HKT1;1 (A,B) and HKT1;4 (C,D) were exposed to 14d of 9 dS m-1. Biomass was significantly reduced by salt treatment in all lines (p < 0.05). Error bars represent standard error of the mean where n = 12–20 plants. Kitaake was used as a WT control in all experiments. (TIF) [file pgen.1006823.s011.tif]

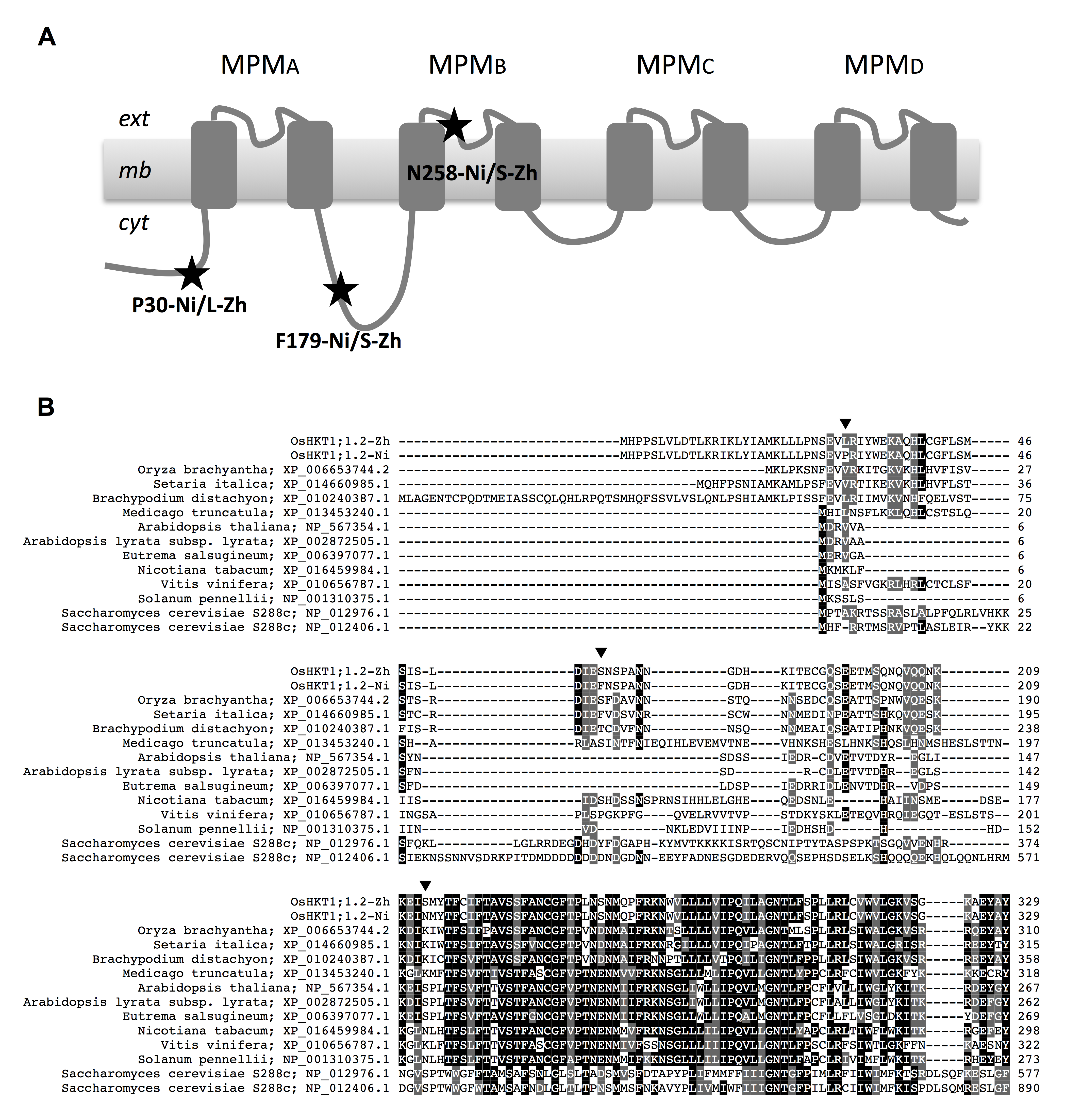

Supplement: S12 Fig — (A) Secondary structure of OsHKT1;1 polypeptide showing the position of AA changes, as exemplified between ‘Nipponbare’ and ‘Zhenshan 2’ variants. (B) Protein alignment of OsHKT1;1.2 and HKT1;1 homologs from various species. Black triangles indicate position of three non-synonymous mutations present in HKT1;1-Zh. (TIF) [file pgen.1006823.s012.tif]

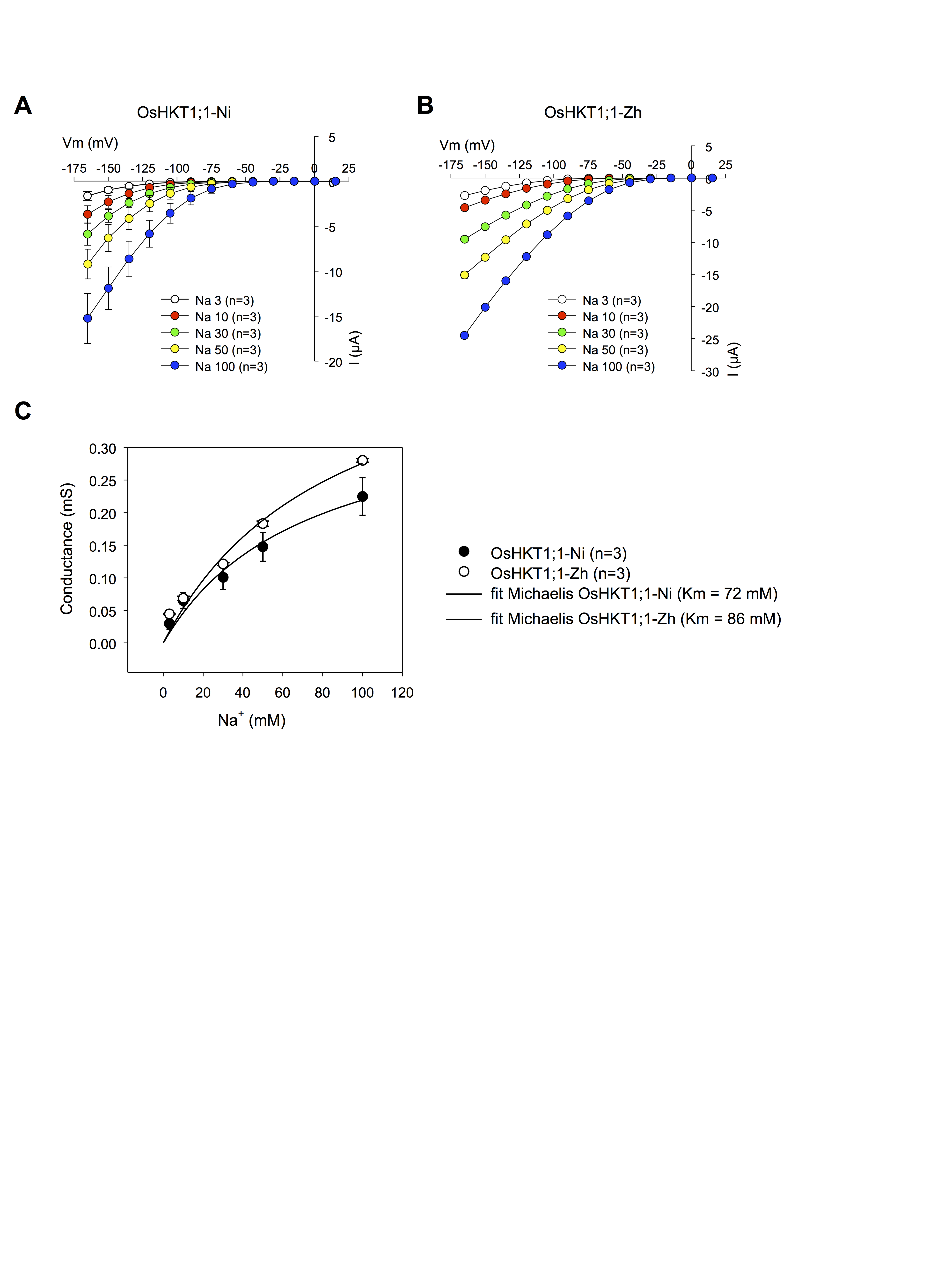

Supplement: S13 Fig — (A, B) Current voltage (I-V) relationships determined in solutions containing 3 to 100 mM Na+-glutamate in HKT1;1-Ni (A) or HKT1;1-Zh (B) expressing oocytes from the same batch. (C) HKT1;1-Ni or -Zh inward conductance plotted versus external Na+ concentration. Whole oocyte HKT1;1 inward conductances, were determined from I-V data shown in (A) and (B) at membrane voltages from -120 to -150 mV. HKT1;1-Ni and–Zh conductances were not significantly different as determined using Student’s t test (p> 0.05). The concentration at which half saturation of the mean conductance occurred (apparent Km) and the maximal conductance were determined with hyperbolic fits (Michaelis-Menten equation): Km = 72 mM and Gmax = 0.38 mS for HKT1;1-Ni, Km = 86 mM and Gmax = 0.51 mS for HKT1;1-Zh. Data in (A-C) are means ± SE. (TIF) [file pgen.1006823.s013.tif]

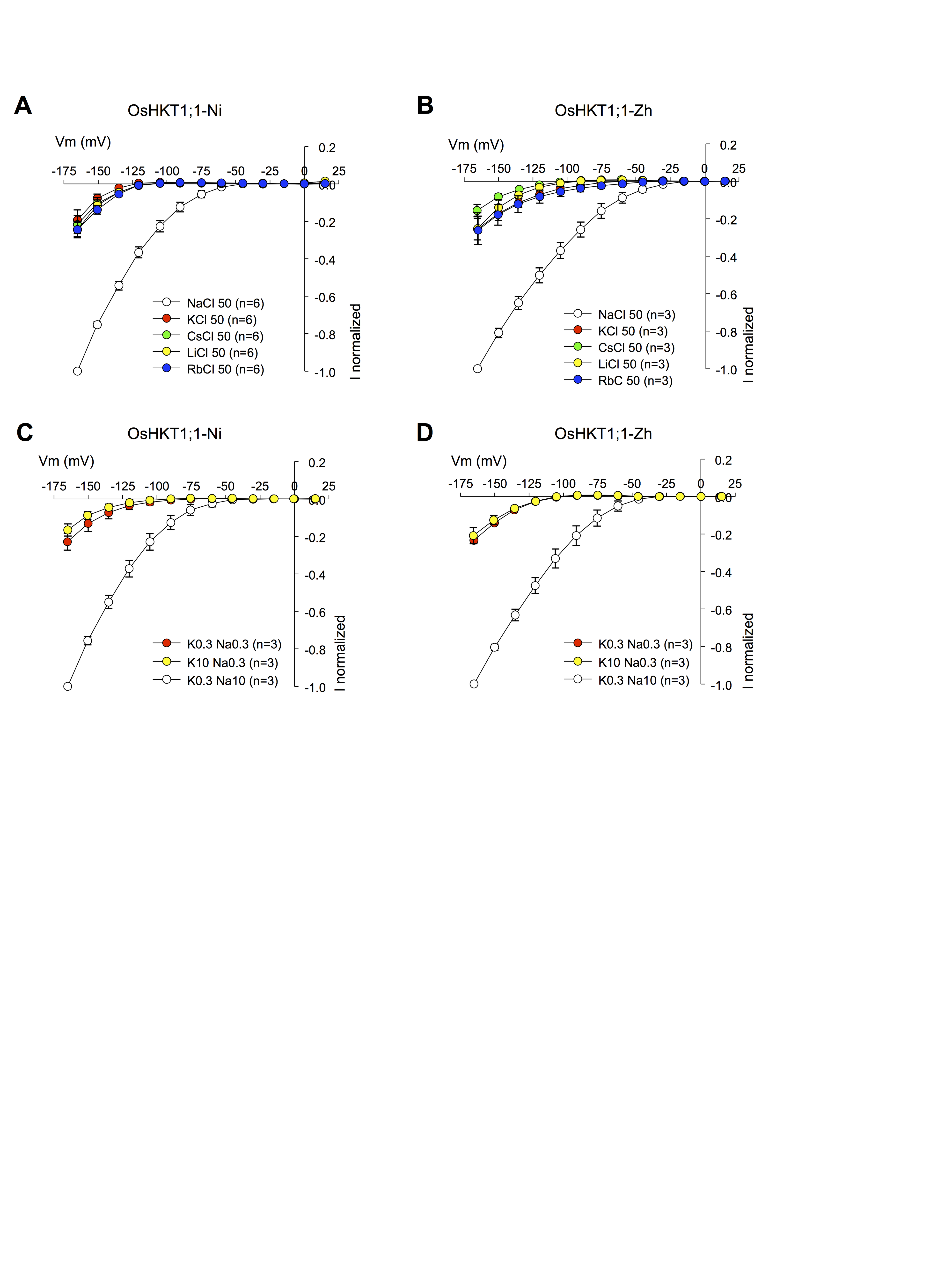

Supplement: S14 Fig — (A-D) Oocytes expressing HKT1;1-Ni (A, C) or HKT1;1-Zh (B, D) were successively bathed with solutions containing 50 mmoles•l-1 of different monovalent cations (Na+, K+, Li+, Rb+, or Cs+, as chloride salts)(A, B) or different combinations of Na+ and K+ concentrations (as glutamate salts)(C, D). Shown I-V relationships were drawn using normalized currents (to those recorded in each oocyte at -165 mV in 50 mM Na+ in (A) and (B), and in 10 mM Na+ and 0.3 mM K+ in (C) and (D)), in order to suppress small differences in expression level between oocytes. Data are means ± SE. (TIF) [file pgen.1006823.s014.tif]

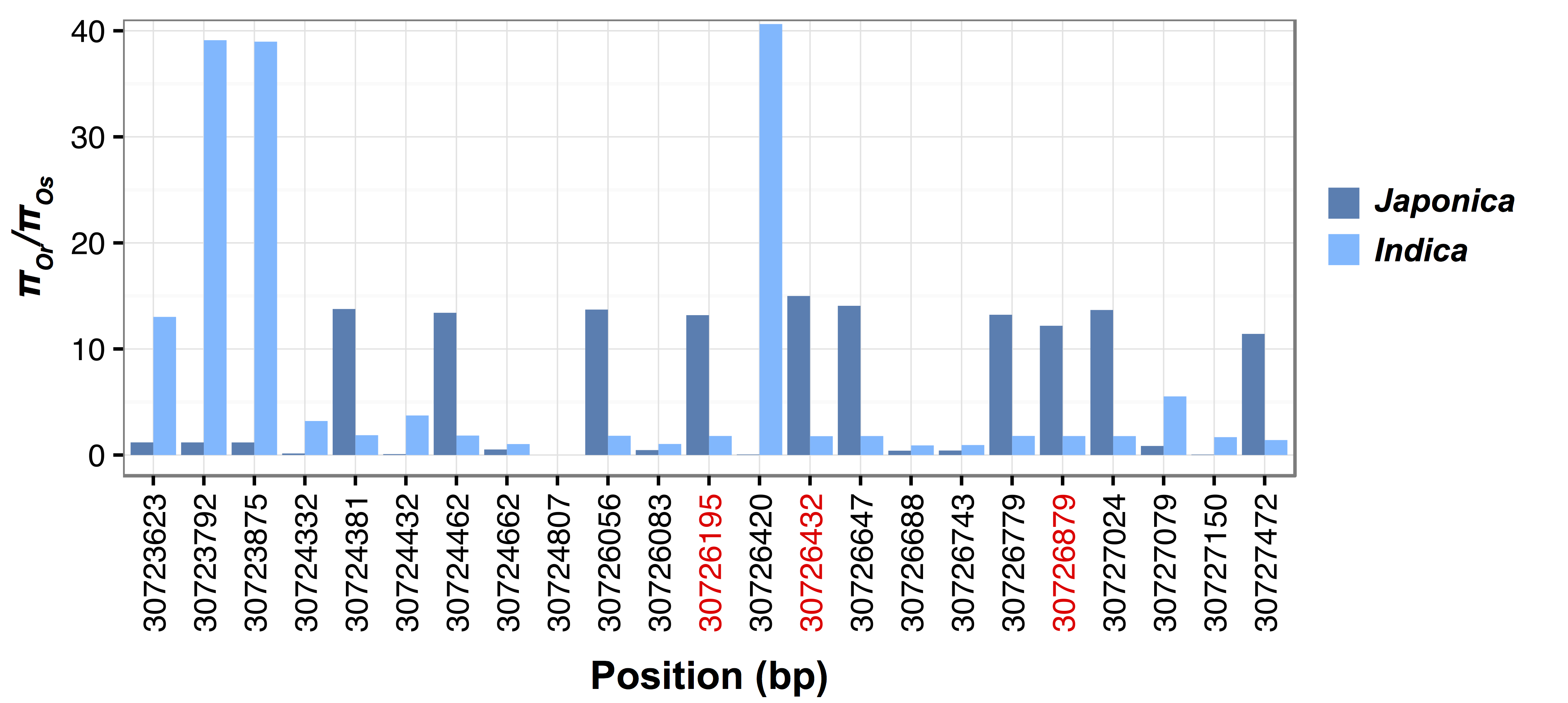

Supplement: S15 Fig — The three non-synonymous SNPs are highlighted in red. The y-axis represents the ratio of the nucleotide diversity (pi) in Oryza rufipogon to Oryza sativa japonica or Oryza sativa indica. (TIF) [file pgen.1006823.s015.tif]
